# Calcium exerts a strong influence upon phosphohydrolase gene abundance and phylogenetic diversity in soil

**DOI:** 10.1016/j.soilbio.2019.107613

**Published:** 2019-12

**Authors:** Andrew L. Neal, Margaret J. Glendining

**Affiliations:** a Department of Sustainable Agriculture Sciences, Rothamsted Research, Harpenden, Hertfordshire, UK; b Computational and Analytical Sciences, Rothamsted Research, Harpenden, Hertfordshire, UK

**Keywords:** Phosphatase, Phytase, Soil, Calcium, Olsen-P, Metagenomics

## Abstract

The mechanisms by which microbial communities maintain functions within the context of changing environments are key to a wide variety of environmental processes. In soil, these mechanisms support fertility. Genes associated with hydrolysis of organic phosphoesters represent an interesting set of genes with which to study maintenance of function in microbiomes. Here, we shown that the richness of ecotypes for each gene varies considerably in response to application of manure and various inorganic fertilizer combinations. We show, at unprecedented phylogenetic resolution, that phylogenetic diversity of phosphohydrolase genes are more responsive to soil management and edaphic factors than the taxonomic biomarker 16S rRNA gene. Available phosphorus – assessed by measuring Olsen-P - exerted some influence on alkaline phosphatase distribution: however, consistent and significant differences were observed in gene abundance between treatments that were inconsistent with bioavailable orthophosphate being the dominant factor determining gene abundance. Instead, we observed gene niche separation which was most strongly associated with soil exchangeable calcium. Our study suggests that the bioavailability of enzyme cofactors (exchangeable calcium in the case of *phoD*, *phoX* and βPPhy studied here) influence the abundance of genes in soil microbial communities; in the absence of cofactors, genes coding for alternative enzyme families that do not require the limiting cofactor (for example, non-specific acid phosphatases which require vanadate) become more abundant.

## Highlights

• Metagenomics-based study of the longest running Long-Term Experiment in the World.
• Studies 16SrRNA-contingent soil communities and nine phosphohydrolase genes.
• Studies the effect of addition of farmyard manure or inorganic fertilizers.
• Demonstrates an important role of bioavailable calcium in gene distribution.
• The influence of bioavailable calcium is greater than bioavailable phosphorus.

## 1 Introduction

Global-scale censuses of soil microbiomes are identifying distinct patterns in the distribution of both bacterial species and community functions. A relatively small subset of bacterial phylotypes dominate soils across the globe (Delgado-Baquerizo et al., 2018) and the taxonomic and gene functional diversity of bacterial assemblages peak at mid-latitudes, declining towards the poles and equator (Bahram et al., 2018). Environmental variables such as climate (aridity, maximum temperature, precipitation characteristics), plant productivity, but especially soil pH (Fierer and Jackson, 2006; Kaiser et al., 2016; Delgado-Baquerizo et al., 2018; Bahram et al., 2018) are more important than dispersal in determining global microbiome species assemblage and functions. The limited number of dominant phylotypes cluster into predictable ecological groups which share similar environmental niches (Delgado-Baquerizo et al., 2018), but habitat preferences are not linked to phylogeny at coarse biological resolution. It is also evident that the functional potential of soil microbiomes is vast, but under-explored: most soil bacterial phylotypes are rare, relatively few abundant (Delgado-Baquerizo et al., 2018). Given this characteristic complex mix of numerous rare and few abundant phylotypes, the mechanisms by which important functions are maintained within microbiomes across ranges of climatic and edaphic factors, especially those brought about by land management or climate change, are both intriguing and not particularly well understood.

Understanding the effects of land management upon soil microbial communities is critical for the provision of a significant number of environmental services including the regulation of biogeochemical cycles and delivery of nutrients to primary producers, degradation of pollutants and provision of clean water, regulation of atmospheric trace gases, and pest and pathogen control (Haygarth and Ritz, 2009; Lehman et al., 2015). Organic compounds (soil organic matter or SOM) are processed by the microbiome using energy derived largely from C in SOM. The accessibility of this energy source depends on its nutritional context in organic matter (since complex bonds may have to be broken to release it) and by the degree to which soil affords the molecules physical protection (in small pores or through chemical bonding to surfaces). Therefore, soil type and the nutritional complexity of inputs affect nutrient cycling rates and the fate of C and other nutrients in soil.

Organic nitrogen (N), sulfur and phosphorus (P) compounds, constituents of SOM, are also degraded and assimilated as nitrate or ammonium, sulfate and orthophosphate respectively. Analogous to C processing, microbial processes play a central role in determining whether N in SOM is released to the atmosphere as N_2_O or N_2_ or retained in the soil. Fertility of soil depends to a large degree upon cycling of complex organic compounds to simple inorganic ions by the soil microbiome. However, modern agriculture has become dependent upon inorganic fertilizer to support ever greater crop yields - often at the expense of nutrient use efficiency and wider environmental pollution. Unlike C or N, P cannot typically be lost from soil to the atmosphere but may be lost *via* run-off to groundwater or surface water bodies where it is the principal cause of eutrophication. The importance of organic phosphorus (P_org_) in the global P-cycle, and the role that bacteria play in its cycling, has interested researchers since the beginning of the 20th century (see Harrison, 1987 for an excellent guide to this literature; Richardson and Simpson, 2011; Haygarth et al., 2018). This is because of its importance in regulating movement of P between terrestrial and freshwater and marine ecosystems and as a potential nutrient source to support plants in natural systems, and particularly, agricultural production.

Thorough descriptions of the biodiversity of genes involved in P_org_ cycling have only recently been published (for the alkaline phosphatases *phoA, phoD* and *phoX*, Sebastian and Ammerman, 2009; Zaheer et al., 2009; Neal et al., 2017 - bacterial non-specific acid phosphatases (NSAP), Gandhi and Chandra, 2012; Neal et al., 2018 - phytases Lim et al., 2007; Neal et al., 2017) and the rest remain poorly described. Collectively, the group of gene families associated with hydrolysis of P_org_-containing compounds represent an interesting set of genes with which to study how microbiomes maintain important functions in the face of environmental change. They all participate in the same process (coding enzymes associated with acquisition of orthophosphate from various P_org_ moieties) so in many respects may be interchangeable, and as genes coding for the catalysis of extracellular nutrients - and so positioned on the periphery of metabolic networks - are likely to be subject to horizontal genetic transfer between individual cells within microbiomes (Pál et al., 2005; Takemoto, 2012). There are clear and consistent links between soil structural parameters and the abundance and diversity of bacterial genes coding for orthophosphate-releasing exoenzymes. In soils having the same texture and exposed to the same climate but of contrasting SOM and connected porosity, genes coding for intracellular phosphatase protein families decrease in abundance in response to reduced pore connectivity while genes coding for protein families of both endo- and exoenzymes (alkaline phosphatases PhoD, PhoX, β-propeller phytase and class C non-specific acid phosphatase) show no reduction in abundance. Furthermore, for the latter protein families, genes predicted to code for exoenzymes are more abundant in poorly structured soil (Neal et al., 2017,Neal et al., 2018). The origin of phosphatases in soils, and the distribution and abundance of genes coding for the various families are not particularly well understood, neither is the effect of fertility management upon gene dynamics (George et al., 2018). There is evidence that several edaphic factors including soil pH (Ragot et al., 2015), organic matter (Sakurai et al., 2008) and fertilizer applications (Chen et al., 2017, 2019; Fraser et al., 2015) affect the distribution and abundance of the principal alkaline phosphatase gene, *phoD*. General land use can also influence the abundance of phosphodydrolase genes (Neal et al., 2018; Liu et al., 2018). A negative association between available P and *phoD* abundance is often, though not exclusively, observed (*e.g*. Chen et al., 2019; Fraser et al., 2015) suggesting that P availability may influence *phoD* gene assemblages directly. A major drawback of our knowledge regarding phosphohydrolase gene distribution in soils is that it is based largely upon amplicon sequencing of *phoD* (Fraser et al., 2015; Ragot et al., 2015, 2017; Chen et al., 2019) or *phoX* (Ragot et al., 2017) genes. This approach relies upon the effectiveness of the primers used to amplify the total diversity of genes within the environment; however even with well-studied genes such as the 16S rRNA gene, this is never the case. For example, in side-by-side comparisons of 16S rRNA-based amplicon description of microbial communities with shotgun metagenome approaches, between 1.5- and 10-times as many phyla and genera were identified in shotgun metagenomes (Poretsky et al., 2014). Unfortunately, the efficacy of primers used in published studies to describe *phoD* and *phoX* distribution are rarely reported and what little evidence is available is not promising. A variety of *phoX* primers investigated by Ragot et al. (2017) were able to amplify only between 3% and 54% of reference sequences *in silico*, suggesting that much of the biodiversity in soil remains undetected, and the extent of this under-representation is unquantifiable. Furthermore, the extent to which diversity of these genes relates to species diversity, typically assessed using the 16S rRNA gene, remains unknown.

To address these issues, we compared the abundance and phylogenetic diversity, using shotgun metagenomics, of a suite of phosphohydrolase genes and the 16S rRNA gene in soil from a long-term field experiment, where soils are amended with farmyard manure or various combinations of inorganic fertilizer. Our aim was to test the hypothesis, established from amplicon sequencing approaches, that phosphohydrolase genes in soil are sensitive to soil management, either by being relatively more abundant in soil receiving farmyard manure because of the greater input of organic residues, or by being relatively more abundant in arable soil that has never received phosphorus fertilizer because of an increased requirement to scavenge phosphorus. Our interest in performing this work was to understand how fertilization of arable soils influences the phylogenetic diversity (or microdiversity) of genes potentially associated with organic P turnover. For this reason, we chose to study the gene sequences themselves, taking advantage of the higher variability of nucleotide sequences to establish relationships between closely related gene sequences that might not be differentiated at the amino acid level. This approach has several advantages over more longstanding approaches: because it does not rely upon amplification of target sequences there is no primer bias and the total gene diversity is equally likely to be identified; since the approach is nucleotide sequence based, phylogenetic relatedness between gene assemblages in different soils can be compared, something traditional diversity measures neglect; by employing rarefaction of estimates of phylogenetic diversity, some assessment is possible regarding the diversity in the environment which remains unaccounted for. These advantages must be balanced with the fact that the method can only identify the known diversity for a given gene, based upon sequenced organisms, that the prediction of a given function is based to a large extent simply upon nucleic acid sequence homology to a limited number of well characterized genes and that only the relative – not absolute – abundance of genes within a community can be measured. Using this approach, we show that the response of phosphohydrolase genes to soil management differs from the response of the 16S rRNA gene, and that individual phosphohydrolase genes occupy distinct niches within the soils, demarcated principally by exchangeable calcium and not soil organic matter or the availability of phosphorus.

## 2 Materials and methods

*Field Experiment and Sampling* - Soil was sampled in October 2015 from four treatments of the Broadbalk Long-Term field experiment (51°48′35″ N, 00°22′30″ W, Johnston and Poulton, 2018). The experimental soil is a clay loam to silty clay loam over clay with flints (FAO Classification: Chromic Luvisol) and is slightly calcareous. The experiment is under-drained and free draining. The four treatment plots are on Section 1 of the experiment, which has been sown continuously with winter wheat (*Triticum aestivum* L., most recently Crusoe seed coated with Redigo® Deter® combination insecticide/fungicide treatment, Bayer CropScience) since 1843, except for occasional fallow years to control weeds. The following four treatment plots were compared: composted farmyard manure (from cattle) applied at a rate of 35 t ha^-1^ per year since 1843 (manure); complete inorganic fertilizer (fertilizer *^+ NP^*) containing 144 kg ha^-1^ nitrogen (N), 35 kg ha^-1^ phosphorus (P) as triple superphosphate (calcium dihydrogen phosphate), 90 kg ha^-1^ potassium (K) as potassium sulfate, and 12 kg ha^-1^ magnesium (Mg) as kieserite per year since 1852; inorganic fertilizer lacking N but receiving 35 kg ha^-1^ P, 90 kg ha^-1^ K and 12 kg ha^-1^ Mg per year (fertilizer*^-N^*) since 1852; inorganic fertilization lacking triple superphosphate but receiving 192 kg ha^-1^ N, (96 kg ha^-1^ N 1906–2000), 90 kg ha^-1^ K and 12 kg ha^-1^ Mg per year since 1906 (fertilizer-P). Nitrogenous fertilizer was applied to fertilizer ^*+ NP*^ and fertilizer*^-P^* soils as ammonium sulfate until 1967, calcium ammonium nitrate (Nitro-chalk) 1968–1985 and as Nitram® ammonium nitrate since 1986. Since 2001, fertilizer *+ NP* and fertilizer^*-N*^ plots have not received P as it was considered in excess. The plough layer (0–23 cm) is limed when necessary - due to increasing soil acidity largely resulting from long-term use of ammonium sulfate as a source of N on some plots - to maintain a minimum soil pH of 7.0–7.5. Liming began in Autumn 1954, and a total chalk application of 18.4 and 10.4 t ha^-1^ was applied to fertilizer *+ NP* and fertilizer^*-P*^ soils respectively, up until 1974. No chalk was applied to the other soils. From 1975 to 1989 a regular scheme was introduced, and a total of 14.7 t ha^-1^ chalk was applied to each soil. No further chalk was required until 2007. Since then, fertilizer *+ NP* soil received a total of 6 t ha^-1^ chalk up to 2015; the other three plots did not require chalk over this period. All soils are tilled conventionally. Since treatments are not replicated on the field experiment, three *pseudo*-replicates were collected from each treatment plot. These *pseudo*-replicates were collected from each end and the centre of the plot, approximately 9 m apart. All sampling equipment was cleaned with 70% ethanol between samples. The top 10 cm of soil was sampled with a 3-cm diameter auger. For each pseudo-replicate, ten cores were pooled and thoroughly mixed whilst sieving through a 2-mm mesh. Samples were then frozen and stored at −80 °C.

Chemical properties of Broadbalk soils have been measured routinely since the experiment inception. Historical data for Olsen-P was taken from the *e*-RA database (Perryman et al., 2018) maintained by Rothamsted Research. Plant-available (Olsen) phosphorus was extracted in 0.5 M sodium bicarbonate before being measured, most recently on a NexION® 300X inductively-coupled plasma mass spectrometer (PerkinElmer LAS (UK) Ltd., Seer Green, UK). Exchangeable potassium (K_ex_), calcium (Ca_ex_), magnesium (Mg_ex_) and sodium (Na_ex_) concentrations were measured on an Optima® inductively-coupled plasma spectrometer (ICP-OES, PerkinElmer) following extraction in a 1 M ammonium acetate solution (pH 7). Total nitrogen (N) was measured by combustion using a Leco® TruMac® analyser (LECO (UK), Stockport, UK) and soil organic carbon (SOC) was measured by ultra-violet oxidation using a TOC-V WP Analyser (Shimadzu UK Ltd., Milton Keynes, UK). Soil pH was measured in water (1:2.5 soil: solution).

*DNA Extraction, Sequencing and Quality Control* - Soil community DNA was extracted from a minimum of 2 g of thawed soil using MoBio PowerSoil® DNA isolation kits (Mo Bio Laboratories, Inc. Carlsbad, CA). DNA quantification and quality control was assessed using a Qubit 2.0 fluorimeter (Thermo Fisher Scientific, Waltham, USA) and 2100 Bioanalyzer DNA chips (Agilent Technologies, Santa Clara, USA). 10 μg of high-quality DNA was provided for sequencing for each of the twelve samples.

Shotgun metagenomic sequencing of DNA was performed using 150 base paired-end chemistry on an Illumina® HiSeq™ 2500 sequencing platform by Beijing Novogene Bioinformatics Technology Co. Ltd. (Beijing, China). The generated raw sequences were limited to a minimum quality score of 25 and a minimum read length of 70 bases using Trimmomatic (Bolger et al., 2014). After filtering to remove substandard sequences, the average number of metagenome reads for each soil was 4.08 × 10^8^ for manure amended soil, 4.37 × 10^8^ for fertilizer^*+NP*^ soil, 3.85 × 10^8^ for fertilizer^*-P*^ soil, and 4.25 × 10^8^ for fertilizer^*-N*^ soil (range across all datasets 3.67 × 10^8^ – 4.61 × 10^8^ reads). Detailed information regarding the number of reads generated for each metagenome dataset, and the number of reads remaining following processing by Trimmomatic are provided in Supplementary Table 1.

*Estimation of gene relative abundance and phylogeny* - Each of the twelve metagenomes generated in this study were analysed to estimate the relative abundance of the 16S rRNA gene and each of nine phosphohydrolase genes. Nucleotide-based profile hidden Markov models (pHMM) were generated from multi-sequence alignments (MSAs) of reference sequences of each gene using hmmbuild, part of the HMMER ver 3.1 suite (Eddy, 2009). MSAs were generated using the *E-INS-i* iterative refinement algorithm in MAFFT version 7.3 (Katoh and Standley, 2013) using the 1PAM/κ = 2 scoring matrix. For the 16S rRNA gene, the pHMM was generated using the set of 4528 reference sequences associated with paprica (Bowman and Ducklow, 2015), built December 2017. For phosphohydrolase genes, pHMMs were generated from reference sequences of the alkaline phosphatase *phoD*, *phoX* and *phoA*, and β-propeller (βPPhy), cysteine (CPhy) and histidine acid (HAPhy) phytase described by Neal et al. (2017), and for classes A, B and C of non-specific acid phosphatase (NSAP) described by Neal et al. (2018). Metagenome reads with homology to the pHMMs were identified using hmmsearch with a 1 × 10^−5^ Expect-value (*E*) cut-off. To allow meaningful comparison between metagenomic datasets, gene relative abundance was expressed as a proportion of the estimated total number of genomes in each dataset, assessed by estimating the abundance of the ubiquitous, single-copy genes *recA*, *gyrB* (Santos and Ochman, 2004) and *atpD* (Gaunt et al., 2001). Nucleotide sequence-based pHMMs were developed for each gene as described in Neal et al. (2017). Metagenome-derived homologue counts for each single-copy gene were size-normalized to the length of the shortest gene pHMM, *recA* accounting for differences in length between the genes. To do this, the pHMM length of *recA* (1164 nt) was divided by the pHMM length of the other single-copy genes (1392 nt for *atpD*, and 2618 nt for *gyrB*), and this value was then multiplied by each single-copy gene count. The length-normalized abundance of each target phosphohydrolase gene was then calculated for each soil as [target gene count·read length/(mean normalized counts of single-copy genes)] (Howard et al., 2008).

PHMMER was used to compare the retrieved metagenome sequences, following six-frame translation using EMBOSS Transeq (Rice et al., 2000), to the UniprotKB protein sequence database to confirm that the sequences represented the correct protein family. Only those metagenome sequences for which one of the six frame translations elicited a UniprotKB hit of the appropriate protein family (*E* < 1 × 10^−5^) was included in the subsequent analysis. Metagenome reads showing homology to each gene were assigned to branches of phylogenetic trees generated from the respective reference gene sets using a phylogenetic placement algorithm, pplacer version 1.1alpha10 (Matsen et al., 2010) and visualized using iTOL version 4.2.3 (Letunic and Bork, 2016). For the 16S rRNA gene, these placements can be translated into robust relative abundance estimates of named organisms using the taxonomic labelling of the tree branches. This is not the case for the phosphohydrolase genes where instead, placement indicates the degree of homology of the metagenome reads (ecotypes) to the respective genes found in sequenced organisms, identified by taxonomic labels of the tree branches. Metagenomes are publicly available at the *e*-RA database (http://www.era.rothamsted.ac.uk/contact) together with comprehensive historical environmental data associated with the soils.

*Statistical Analysis* – To test our hypotheses, we generated several gene assemblage-related metrics, including relative abundance, phylogenetic diversity and phylogeny-based distance metrics. The effects of different fertilizer treatments upon edaphic factors and estimates of normalized relative abundance and α-diversity for each gene were analysed using analysis of variance (ANOVA) after testing for homogeneity of variances using Levene's test and normality using the Shapiro-Wilk test. Data for some genes were associated with significantly non-normal distributions, although the variances were homogenous. Permutation-based distribution-free tests of significance of *F*-values were therefore adopted to calculate probability (denoted as *p*_perm_). Where significant treatment effects were identified, *post-hoc* pair-wise comparisons were performed using Tukey-Kramer Studentized *Q*, following the Copenhaver-Holland multiple comparison procedure (Copenhaver and Holland, 1988). All tests were calculated using PAST version 3.2 (Hammer et al., 2001). For all tests, an α of 0.05 was considered significant.

Estimates of gene phylogenetic (that is, sequence similarity-sensitive) diversity based upon placement of homologous metagenomic reads were assessed by computing a measure incorporating abundance, balance-weighted phylogenetic diversity (BWPD_1_, McCoy and Matsen, 2013) using the guppy fpd binary (part of the pplacer code), accounting for pendant branch length. To assess the depth of sequencing of the soil communities compared with the total diversity of the nine genes within them, rarefaction curves of expected mean phylogenetic diversity (Nipperess and Matsen, 2013) were generated using the guppy rarefact binary, interpreting placement weights as counts and calculating up to a rarefaction size (*k*) of 70,000. Additionally, unconstrained ordination based upon principal component analysis of the difference in placement densities on reference tree branches, termed edge-PCA (Matsen and Evans, 2013), was used for graphical representation of phylogeny-based differences between treatments in a two-dimensional plane using the guppy epca binary and treating each query as a point mass concentrated on the highest-weight placement. One advantage of edge-PCA is that branches associated with placements contributing to eigenvalues on each axis are identified and for 16S rRNA analysis, organisms contributing to the observed differences can be identified. However, this is not the case for other genes where only association with sequenced homologs can be identified. We therefore made no attempt to infer the likely organisms associated with the various PHO genes in the soils.

To assess 16S rRNA and PHO gene-based β-diversity in the different soils, Kantorovich-Rubinstein (KR) phylogenetic distance metrics (Evans and Matsen, 2012) were calculated from phylogenetic placements of metagenome reads using the guppy kr binary, again treating each query as a point mass concentrated on the highest-weight placement. The advantage of the KR distance metric is that it compares gene assemblage distributions on a phylogenetic tree (of 16S rRNA or other genes), in units of nucleotide substitutions per site, and is therefore a biologically meaningful approach to comparing communities. Differences in gene assemblages based upon KR metrics were tested using permutational multivariate analysis of variance (PERMANOVA, Anderson and ter Braak, 2003) following testing for homogeneity of multivariate dispersions among *a priori* groups using PERMDISP (Anderson, 2006). These tests were performed using PRIMER PERMANOVA + ver 7.0.13 (PRIMER-e, Auckland, New Zealand). Where no significant heterogeneity of multivariate dispersion was detected, pair-wise comparisons were performed, however since the number of observations was insufficient to allow a reasonable number of permutations, Monte Carlo probabilities (denoted *p*_MC_) were calculated based upon an asymptotic permutation distribution (Anderson and Robinson, 2003).

To model the contribution of edaphic factors to observed phylogenetic distributions, where significant differences in phylogeny between soils were detected by PERMANOVA, we employed distance-based redundancy analysis (dbRDA, Anderson and Legendre, 1999) of KR metrics. In this approach, multivariate multiple regression of principal coordinate axes on predictor variables is used to identify linear combinations of those predictor variables which explain the greatest variation in a multivariate dataset. Since the analysis employs KR distance directly, the ordinations can be interpreted as the phylogenetic response (in units of substitutions per site) of the communities to the predictor variables. Edaphic factors, listed in Table I, were employed as potential predictor variables and were selected according to which were best in explaining the variation in treatments. The small-sample corrected Akaike Information Criterion (AIC_c_) was used to identify the best combination of at least two variables to describe the observed distribution of treatments. These steps were performed in PRIMER PERMANOVA+ and were based upon 99,999 permutations.

**Table 1:** Edaphic parameters for plots of the Broadbalk winter wheat long-term experiment used in this study. The mean and standard error of estimates are shown for each treatment (*n* = 3, measured in 2000, 2005 and 2010). Exchangeable cations (K_ex_, Ca_ex_, Mg_ex_ and Na_ex_) were estimated following extraction in ammonium acetate, total nitrogen by combustion, SOC by ultra-violet oxidation, pH was measured in water (1:2.5 soil: solution). Treatment effects upon the different parameters were tested using either parametric analysis of variance (where an *F* statistic is provided) or non-parametric Kruskal-Wallace test (where an *H* statistic is provided) where data distributions did not meet the assumptions of ANOVA following transformation. Where significant treatment effects are detected, superscripted letters indicate significant differences between treatment means, established by Tukey-Kramer pairwise comparisons (α = 0.05).

	pH	SOC/%	Nitrogen/%	C/N ratio	Olsen-P/mg P kg^-1^	K_ex_/mg kg^-1^	Ca_ex_/g kg^-1^
	*H* = 6.3, *p* = 0.096	*F*_3,8_ = 528, *p* < 0.001	*F*_3,8_ = 308, *p* < 0.001	*F*_3,8_ = 3.19, *p* = 0.084	*F*_3,8_ = 218, *p* < 0.001	*F*_3,8_ = 82.1, *p* < 0.001	*F*_3,8_ = 75.1, *p* < 0.001
Manure amended	7.8 ± 0.05	2.9 ± 0.07^a^	0.28 ± 0.008^a^	10.4 ± 0.15	96.7 ± 3.5^a^	610 ± 24.7^a^	6.1 ± 0.25^a^
Fertilizer *^+ NP^*	7.1 ± 0.4	1.1 ± 0.03^b^	0.11 ± 0.003^b^	9.6 ± 0.29	72.0 ± 2.5^b^	312 ± 4.9^c,d^	2.8 ± 0.04^c^
Fertilizer*^-N^*	8.1 ± 0.06	0.9 ± 0.01^c^	0.095 ± 0.003^b^	9.4 ± 0.24	87.7 ± 3.8^a^	423 ± 10.7^b,c^	5.1 ± 0.14^b^
Fertilizer*^-P^*	8.2 ± 0.04	1.1 ± 0.03^b^	0.11 ± 0.004^b^	9.5 ± 0.29	2.7 ± 0.3^c^	374 ± 7.5^c^	6.6 ± 0.28^a^

	Mg_ex_/mg kg^-1^	Na_ex_/mg kg^-1^
	*F*_3,8_ = 528, *p* < 0.001	*F*_3,8_ = 0.67, *p* = 0.592
Manure amended	117 ± 2.7a	15.3 ± 5.0
Fertilizer ^*+ NP*^	93 ± 1.5^b^	10.7 ± 1.7
Fertilizer^*-N*^	79 ± 0.9^c^	10.0 ± 2.3
Fertilizer^*-P*^	80 ± 2.2^c^	11.0 ± 1.2

## 3 Results

*Soil chemistry and phosphorus concentrations in Broadbalk soils* – We compared four treatments on the Broadbalk winter wheat long-term experiment whose fertility is managed in contrasting ways. Mean estimates of soil parameters are shown in Table 1. Significant treatment effects were observed for most parameters: only soil pH (which is adjusted by application of calcium carbonate to maintain soil pH at a level which does not limit wheat yield), C/N ratio and exchangeable sodium (Na_ex_) concentration showed no statistically significant differences associated with fertility management. The highest concentrations of SOC (2.9%), N (0.28%), exchangeable potassium (K_ex_) (610 mg kg^−1^) and exchangeable magnesium (Mg_ex_) (117 mg kg^−1^) were recorded in manure amended soil. Higher values were observed for fertilizer^*-P*^ in the case of exchangeable calcium (Ca_ex_) (6.6 g kg^−1^), however there was no statistically significant difference between this soil and manure amended soil (6.1 g kg^1^).

**Fig. 1:**
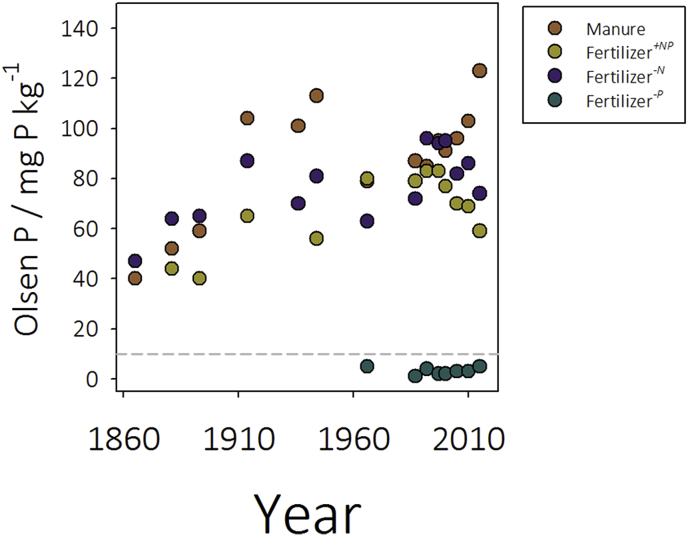
**Olsen-P in soils of the Broadbalk winter wheat long-term experiment.** Sodium bicarbonate extractable (Olsen) phosphorus in soils historically receiving farmyard manure, inorganic fertilizer (NPKMg, fertilizer^*+NP*^), inorganic fertilizer with no nitrogen addition (fertilizer^*-N*^) and inorganic fertilizer with no phosphorus addition (fertilizer^*-P*^). Dashed line indicates the estimated Olsen-P of the soil prior to establishment of the experiment in 1856. Addition of phosphorus was halted in 2000 to fertilizer^*+NP*^ and fertilizer^*-N*^ soils.

Olsen-P concentrations have been recorded in the soils since 1865. Estimated Olsen-P in the original soils was low, at approximately 10 mg P kg^−1^, based upon measurements made on near-by plots in 1856. Up until 2000, Olsen-P increased progressively in manure amended, fertilizer^*+NP*^ and fertilizer^*-N*^ plots (Fig. 1) to over 80 mg P kg^−1^. At this point a decision was taken to cease additions of triple superphosphate fertilizer to the fertilizer^*+NP*^ and fertilizer^*-N*^ treatments with the result that Olsen-P in these soils has reduced consistently year on year. Measurement of Olsen-P on the fertilizer^*-P*^ soil was only instigated in 1966 but has remained consistently below the estimated starting Olsen-P of 10 mg P kg^−1^. The highest Olsen-P concentrations of 97 mg P kg^−1^ are observed in manure amended soil: the least, 3 mg P kg^−1^, in fertilizer^*-P*^ soil.

**Fig. 2:**
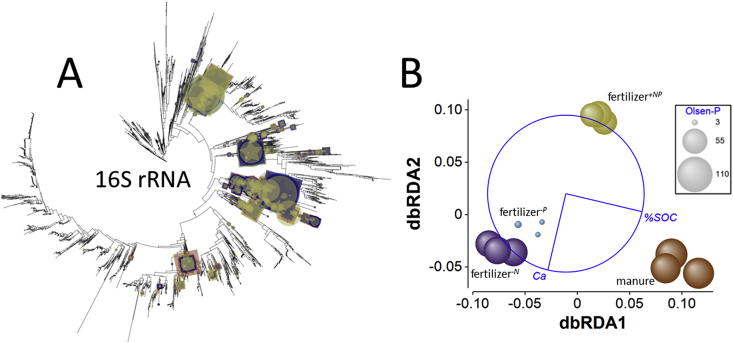
**Effects of soil fertility management upon the microbial assemblages in soil according to 16S rRNA homology**. A – phylogenetic placement of metagenome reads with homology to the bacterial 16S rRNA gene in Broadbalk soils receiving farmyard manure, inorganic fertilizer (NPKMg, fertilizer^*+NP*^), inorganic fertilizer with no nitrogen addition (fertilizer^*-N*^) and inorganic fertilizer with no phosphorus addition (fertilizer^*-P*^). Placement symbol size is scaled to reflect normalized relative abundance across the twelve samples. Different placement shapes – circle, square, star - represent replicates within each treatment. B – Kantorovich-Rubinstein distance-based RDA ordination and partial correlations of %SOC and exchangeable calcium (Ca). Kantorovich-Rubinstein distances are based upon abundance weighted phylogenetic placement of reads shown in A. The data points are scaled to reflect soil Olsen-P (mg P kg^−1^, see Table 1). Model *r*^2^ = 0.677 with dbRDA1 accounting for 58.7% of the fitted and 39.7% of the total variation and dbRDA2 accounting for 41.3% of the fitted and 28.0% of total variation. Multiple partial correlations: dbRDA1 %SOC *r* = 0.973, Ca_ex_*r* = −0.230; dbRDA2 Ca_ex_*r* = −0.973, %SOC = −0.230. The length and direction of each vector indicates the strength and direction of increase, respectively, of the relationship between that variable and the dbRDA axes. The circle is a unit circle (radius = 1.0), the relative size and position of its origin (centre) is arbitrary with respect to the underlying plot. The colours used to differentiate different treatments in B are consistent with A. (For interpretation of the references to colour in this figure legend, the reader is referred to the Web version of this article.)

*Community response to treatments based upon 16S rRNA gene assemblage* – Rarefaction of phylogenetic diversity (the sum of lengths of branches in a phylogenetic tree associated with metagenomic reads) based upon the 16S rRNA gene (Supplementary Appendix Fig. S1A) indicated that in no case was the complete 16S rRNA gene diversity captured, but the extent of diversity accounted for by sequencing was consistent between treatments. No significant differences were detected in balance-weighted phylogenetic diversity (BWPD_1_) of the molecular marker 16S rRNA gene between treatments. However, edge-PCA (Supplementary Appendix, Fig. S1B) showed clear separation of treatments. There was no significant heterogeneity of multivariate dispersion between treatments (*pseudo-F* = 1.1, *p*_perm_ = 0.659), but a significant treatment effect upon 16S rRNA gene phylogeny in the soils (*pseudo*-*F* = 7.2, *p*_perm_ = 0.0002). Pair-wise tests indicated that only the fertilizer^−*N*^ and fertilizer^*-P*^ soils were not significantly different form each other (*pseudo-t* = 1.1, *p*_MC_ = 0.306). The primary edge-PCA axis separated manure amended soil from soils receiving inorganic fertilizer, particularly fertilizer^*-N*^ and fertilizer^*-P*^ soils. On this axis, organisms such as the δ-proteobacteria *Haliangium ochraceum* and *Steroidobacter denitrificans*, the verrucomicrobium *Candidatus* Xiphinematobacter sp. and the planctomycetes *Gemmata* sp. and *Phycisphaera mikurensis* were more abundant in manure amended soil while actinobacterium *Conexibacter woesei*, the chloroflexi *Caldilinea aerophila* and *Sphaerobacter thermophilus*, and *Gemmatimonas aurantiaca* and the closely related *G. phototrophica* were more abundant in fertilizer^*-N*^ and fertilizer^*-P*^ soils. On the second axis, fertilized^*+NP*^ soil was separated from all other treatments. *Ca*. Xiphinematobacter sp. was more abundant in fertilizer^*+NP*^ soil, while *C. aerophila*, *S. denitrificans*, *Gemmata* sp. and *P. mikurensis* all had reduced abundance. These placements and differences in the abundance of each placement can be seen in Fig. 2A. A combination of %SOC and Ca_ex_ was identified by dbRDA as the best combination of variables explaining the distribution of treatments based upon 16S rRNA KR distance metrics. The constrained ordination is shown in Fig. 2B and accounts for 68% of the total variation. Separation of treatments on the principal axis was largely determined by differences in %SOC (accounting for 59% of the fitted variation), separating manure amended from fertilizer amended soils. The second axis was associated with differences in Ca_ex_, separating the manure amended, fertilized^*-N*^ and fertilized^*-P*^ soils with high Ca_ex_ from fertilizer^*+NP*^ soil, which was associated with a low Ca_ex_ and accounting for 41% of the fitted variation.

*Phosphohydrolase gene phylogenetic diversity and response to soil fertilization* – The number of reads identified as *gyrB*, *recA*, and *atpD*, together with the number of reads and relative abundance of each PHO gene, are presented in Supplementary Table 2. Comparison of rarefaction curves generated for each gene (Supplementary Appendix, Fig. S2) indicated that in no case was the genetic diversity in the sampled communities represented completely. However, for each gene the extent of diversity accounted for by sequencing was comparable. These curves also demonstrated distinct differences in the relative abundance and phylogenetic diversity of the different genes. Alkaline phosphatase genes *phoD* and *phoX* were the most abundant and diverse phosphohydrolase genes in the soils. The two classes of NSAP studied presented similar phylogenetic diversity, although class C genes were more abundant. The remaining genes – the alkaline phosphatase *phoA* and the three phytase classes - all showed low diversity: only the β-propeller phytase (βPPhy) gene matched the relative abundance of the NSAP genes.

In contrast to shifts in microbial communities in response to soil fertilization evident from 16S rRNA phylogeny, where the dominant difference was in response to organic inputs from cattle manure *versus* inorganic fertilization, for most genes associated with phosphorus acquisition the major difference was between fertilizer^*+NP*^ soil and the other treatments. This was most evident for the alkaline phosphatase *phoD* and class A NSAP. Also, except for acidic phytase genes, significant differences in BWPD_1_ were identified for phosphohydrolase genes (Supplementary Appendix Fig. S3).

**Fig. 3:**
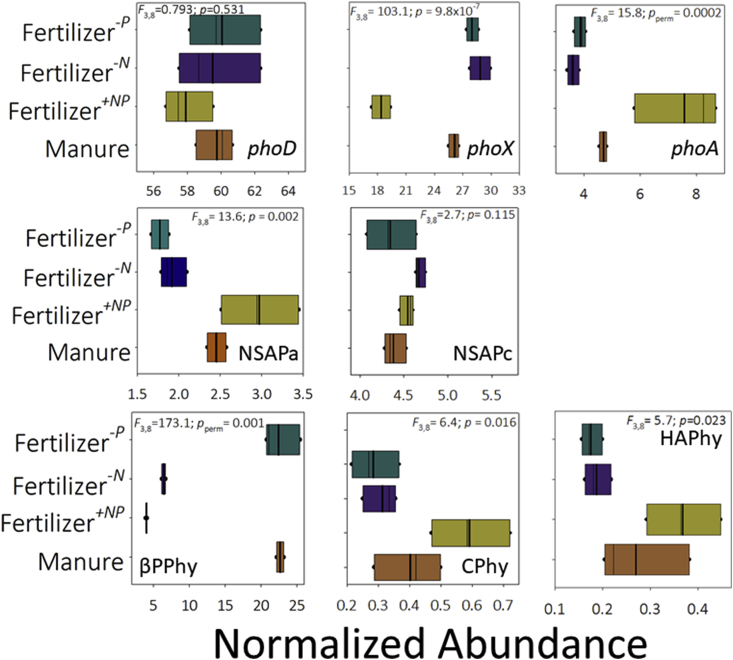
Abundance of phosphohydrolase genes in broadbalk soil. Length-normalized abundance (relative to the three single-copy genes, see materials and methods section for calculation description) of gene ecotypes in soils receiving farmyard manure, inorganic fertilizer (npkmg, fertilizer + np), inorganic fertilizer with no nitrogen addition (fertilizer-n) and inorganic fertilizer with no phosphorus addition (fertilizer-p).

**Fig. 4:**
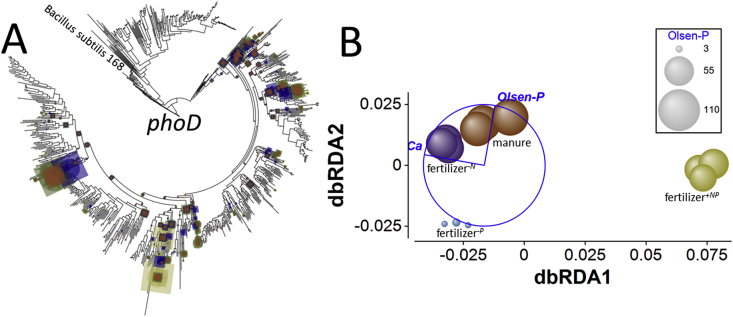
**Effects of soil fertility management upon alkaline phosphatase *phoD* ecotype assemblages in soil.** A – phylogenetic placement of metagenome reads with homology to the *phoD* gene in Broadbalk soils receiving farmyard manure, inorganic fertilizer (NPKMg, fertilizer^*+NP*^), inorganic fertilizer with no nitrogen addition (fertilizer^*-N*^) and inorganic fertilizer with no phosphorus addition (fertilizer^*-P*^). Placement symbol size is scaled to reflect normalized relative abundance across the twelve samples. Different placement shapes – circle, square, star - represent replicates within each treatment. The label refers to the SWISS-PROT *phoD* accession. B – Kantorovich-Rubinstein distance-based RDA ordination and partial correlations of exchangeable calcium (Ca) and Olsen-P. Kantorovich-Rubinstein distances are based upon phylogenetic placement of reads shown in A. The data points are scaled to reflect soil Olsen-P (mg P kg^−1^, see Table 1). Model *r*^2^ = 0.752 with dbRDA1 accounting for 88.6% of the fitted and 66.6% of the total variation and dbRDA2 accounting for 11.4% of the fitted and 8.5% of total variation. Multiple partial correlations: dbRDA1 Ca_ex_*r* = −0.984, Olsen-P *r* = 0.176; dbRDA2 Ca_ex_*r* = 0.176, Olsen-P = 0.984. The length and direction of each vector indicates the strength and direction of increase, respectively, of the relationship between that variable and the dbRDA axes. The circle is a unit circle (radius = 1.0), the relative size and position of its origin (centre) is arbitrary with respect to the underlying plot. The colours used to differentiate different treatments in B are consistent with A. (For interpretation of the references to colour in this figure legend, the reader is referred to the Web version of this article.)

*Alkaline phosphatase genes* – No significant treatment effect on *phoD* normalized relative abundance was detected (Fig. 3), but relative abundance was least in fertilizer^*+NP*^ soil. However, gene BWPD_1_ (Supplementary Appendix Fig. S3) was significantly greater in fertilizer^+*NP*^ soil than under the other treatments (smallest difference, *Q* = 5.7; *p*_perm_ = 0.016). Genes in manure amended soil were also significantly more diverse than in fertilizer^*-P*^ soil (*Q* = 4.9; *p*_perm_ = 0.033). These differences were evident in edge-PCA ordination (Supplementary Appendix Fig. S4) of *phoD* phylogenetic placement (Fig. 4A). Differences between fertilizer^*+NP*^ and the other soils were distributed on the primary edge-PCA axis. The was no significant heterogeneity of multivariate dispersion (*pseudo-F* = 4.3, *p*_perm_ = 0.187). PERMANOVA identified a significant difference between soils (*pseudo-F* = 10.9, *p*_perm_ = 0.0001) and pair-wise comparison indicated that the *phoD* assemblage in fertilizer^*+NP*^ soil was significantly different from the other treatments (smallest difference, *pseudo-t* = 3.9, *p*_MC_ = 0.0038). All other comparisons were significant except for manure amended – fertilizer^*-P*^ soils (*pseudo-t* = 2.3, *p*_MC_ = 0.065) and fertilizer^*-N*^ – fertilizer^*-P*^ soils (*pseudo-t* = 1.3, *p*_MC_ = 0.176). These relationships are clearly seen in the constrained dbRDA ordination (Fig. 4B). Ca_ex_ and Olsen-P were identified as the best combination of environmental variables describing treatment separation, accounting for 75% of total variation. The primary axis separated fertilizer^*+NP*^ soil from the other treatments and is associated with differences in Ca_ex_, which was high in the manure amended, fertilizer^*-N*^ and fertilizer^*-P*^ soils and low in fertilizer^*+NP*^ soil. The second axis effectively separated manure amended soil from fertilizer amended soil in the basis of Olsen-P, which was highest in manure amended soil. This suggests that Ca_ex_ (accounting for 89% of fitted variation) exerts a greater influence upon *phoD* phylogeny than P bioavailability, expressed as Olsen-P (accounting for 11%).

**Fig. 5:**
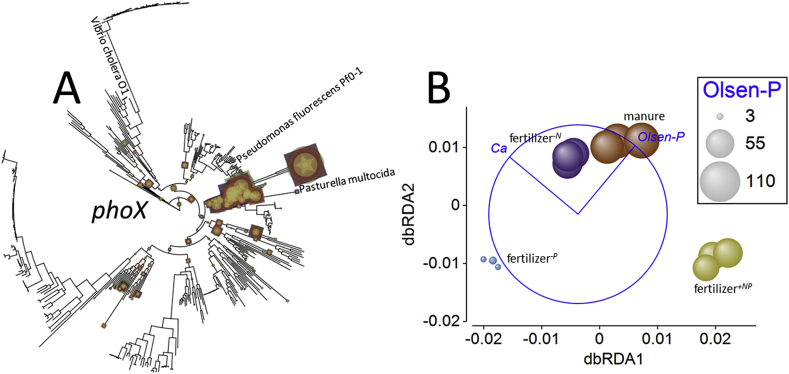
**Effects of soil fertility management upon alkaline phosphatase *phoX* ecotype assemblages in soil.** A – phylogenetic placement of metagenome reads with homology to the *phoX* gene in Broadbalk soils receiving farmyard manure, inorganic fertilizer (NPKMg, fertilizer^*+NP*^), inorganic fertilizer with no nitrogen addition (fertilizer^*-N*^) and inorganic fertilizer with no phosphorus addition (fertilizer^*-P*^). Placement symbol size is scaled to reflect normalized relative abundance across the twelve samples. Different placement shapes – circle, square, star - represent replicates within each treatment. Labels refer to genes described by Majumdar et al. (2005), Wu et al. (2006), and Monds et al. (2006). B – Kantorovich-Rubinstein distance-based RDA ordination and partial correlations of exchangeable calcium (Ca) and Olsen-P. Kantorovich-Rubinstein distances are based upon phylogenetic placement of reads shown in A. The data points are scaled to reflect soil OlsenP (mg P kg^−1^, see Table 1). Model *r*^2^ = 0.387 with dbRDA1 accounting for 68.5% of the fitted and 26.5% of the total variation and dbRDA2 accounting for 31.5% of the fitted and 12.2% of total variation. Partial correlations: dbRDA1 Ca_ex_*r* = −0.768, Olsen-P *r* = 0.641; dbRDA2 Ca_ex_*r* = 0.641, Olsen-P = 0.768. The length and direction of each vector indicates the strength and direction of increase, respectively, of the relationship between that variable and the dbRDA axes. The circle is a unit circle (radius = 1.0), the relative size and position of its origin (centre) is arbitrary with respect to the underlying plot. The colours used to differentiate different treatments in B are consistent with A. (For interpretation of the references to colour in this figure legend, the reader is referred to the Web version of this article.)

The response of *phoX* to the different soil fertility management was distinctly different from that of *phoD*. In this case, both gene normalized relative abundance and BWPD_1_ (Fig. 3 and Supplementary Appendix Fig. S3) were significantly different across the treatments and lowest in fertilizer^*+NP*^ soil. Normalized relative abundance in fertilizer^*+NP*^ soil was significantly lower than for the other soils (smallest difference, *Q* = 16.5; *p* = 1.2 × 10^−5^). The *phoX* normalized relative abundance in manure amended soil was also significantly greater than in fertilizer^*-N*^ soil (*Q* = 5.8; *p* = 0.015). Phylogenetic diversity in fertilizer^*+NP*^ soil was significantly reduced compared to diversity in the fertilizer^*-P*^ soil (*Q* = 5.9; *p*_perm_ = 0.014). No other differences in phylogenetic diversity were significant. Edge-PCA ordination (Supplementary Appendix Fig. S5) based upon phylogenetic placement of metagenome reads (Fig. 5A) showed less consistency within treatments than was evident for *phoD*. No significant heterogeneity of multivariate dispersion was detected (*pseudo-F* = 4.6, *p*_perm_ = 0.126), but significant differences between treatments were identified by PERMANOVA (*pseudo-F* = 2.5, *p*_perm_ = 8 × 10^−5^) however, pair-wise comparison of treatments failed to identify a significant difference (largest difference, fertilizer^*+NP*^ – fertilizer^*-P*^, *pseudo-t* = 1.9, *p*_MC_ = 0.052). The *phoX* assemblages in these soils appeared most dissimilar in the resulting dbRDA ordination (Fig. 5B) where Ca_ex_ and Olsen-P were again identified as the best combination of environmental parameters to describe treatment separation, accounting for 39% of total variation. Again, Ca_ex_ was associated with the primary axis which separated fertilizer^*+NP*^ soil from the other soils and accounted for 69% of fitted variation. The secondary axis was associated again with Olsen-P, separating fertilizer^*-P*^ and fertilizer^*+NP*^ soils from manure amended and fertilizer^*-N*^ soils. Olsen-P accounted for 32% of fitted variation and so as with *phoD*, the effect of Ca_ex_ upon gene phylogeny was greater than Olsen-P.

**Fig. 6:**
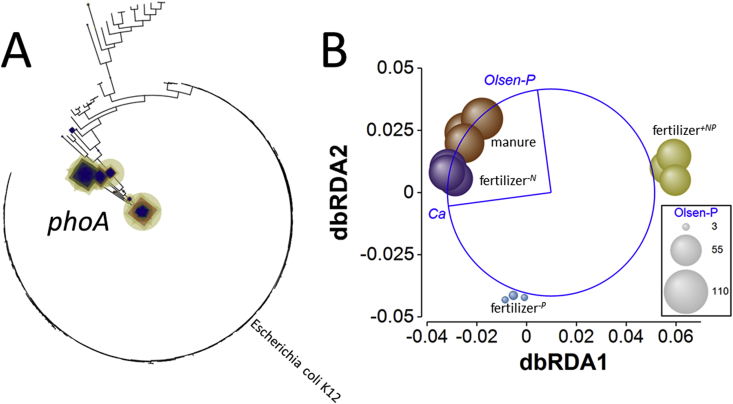
**Effects of soil fertility management upon alkaline phosphatase *phoA* ecotype assemblages in soil.** A – phylogenetic placement of metagenome reads with homology to the *phoA* gene in Broadbalk soils receiving farmyard manure, inorganic fertilizer (NPKMg, fertilizer^*+NP*^), inorganic fertilizer with no nitrogen addition (fertilizer^*-N*^) and inorganic fertilizer with no phosphorus addition (fertilizer^*-P*^). Placement symbol size is scaled to reflect normalized relative abundance across the twelve samples. Different placement shapes – circle, square, star - represent replicates within each treatment. The label refers to the SWISS-PROT *phoA* accession. B – Kantorovich-Rubinstein distance-based RDA ordination and partial correlations of exchangeable calcium (Ca) and Olsen-P. Kantorovich-Rubinstein distances are based upon phylogenetic placement of reads shown in A. The data points are scaled to reflect soil Olsen-P (mg P kg^−1^, see Table 1). Model *r*^2^ = 0.420 with dbRDA1 accounting for 65.5% of the fitted and 27.5% of the total variation and dbRDA2 accounting for 34.5% of the fitted and 14.5% of total variation. Multiple partial correlations: dbRDA1 Ca_ex_*r* = −0.991, Olsen-P *r* = −0.131; dbRDA2 Ca_ex_*r* = −0.131, Olsen-P = 0.991. The length and direction of each vector indicates the strength and direction of increase, respectively, of the relationship between that variable and the dbRDA axes. The circle is a unit circle (radius = 1.0), the relative size and position of its origin (centre) is arbitrary with respect to the underlying plot. The colours used to differentiate different treatments in B are consistent with A. Different placement shapes in A represent replicates within each treatment. (For interpretation of the references to colour in this figure legend, the reader is referred to the Web version of this article.)

For the *phoA* gene, significant treatment effects were evident for both normalized relative abundance and BWPD_1_ (Fig. 3 and Supplementary Fig. S3). In contrast to *phoD* and *phoX*, relative abundance was significantly greater in fertilizer^*+NP*^ soil than any other treatment (smallest difference, *Q* = 8.7, *p* = 0.0013) and this soil also presented significantly lower BWPD_1_ than the other soils (smallest difference, *Q* = 7.0, *p* = 0.005). Edge-PCA ordination showed limited clustering according to treatment (Supplementary Appendix Fig. S6) based upon phylogenetic placement (Fig. 6A). There was no significant heterogeneity of multivariate dispersion (*pseudo-F* = 1.8, *p*_perm_ = 0.329) but a significant difference in *phoA*-based KR distance metrics (*pseudo-F* = 2.7, *p*_perm_ = 0.010). Pair-wise comparisons indicated that only the fertilizer^*+NP*^ – fertilizer^*-N*^ difference was significant (*pseudo-t* = 2.1, *p*_MC_ = 0.040). Again, Ca_ex_ and Olsen-P were identified as the best variables to describe the resulting constrained dbRDA (Fig. 6B), accounting for 42% of total variation. A similar distribution of treatments was observed as for *phoD* and *phoX*, where fertilizer^*+NP*^ soil was separated from the other soils on the principal axis, associated with Ca_ex_ (and accounting for 66% of fitted variation), while the second axis separated fertilizer^*-P*^ soil from the other soils based upon Olsen-P (accounting for 35% of fitted variation).

**Fig. 7:**
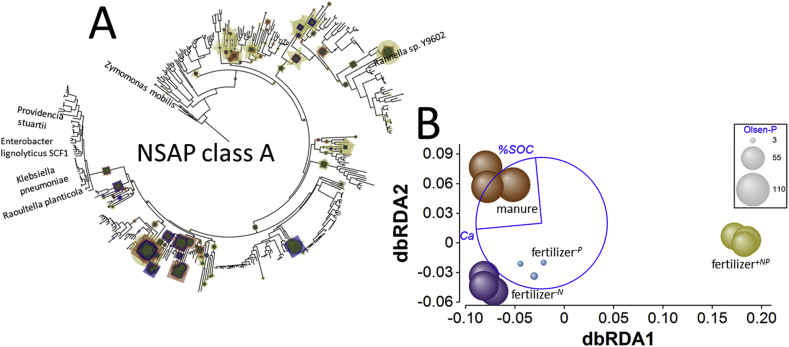
**Effects of soil fertility management upon Class A non-specific acid phosphatase ecotype assemblages in soil.** A – phylogenetic placement of metagenome reads with homology to the nonspecific acid phosphatase class A gene in Broadbalk soils receiving farmyard manure, inorganic fertilizer (NPKMg, fertilizer^*+NP*^), inorganic fertilizer with no nitrogen addition (fertilizer^*-N*^) and inorganic fertilizer with no phosphorus addition (fertilizer^*-P*^). Placement symbol size is scaled to reflect normalized relative abundance across the twelve samples. Different placement shapes – circle, square, star - represent replicates within each treatment. Labels refer to genes described by Gandhi and Chandra (2012). B – Kantorovich-Rubinstein distance-based RDA ordination and partial correlations of exchangeable calcium (Ca) and Olsen-P. Kantorovich-Rubinstein distances are based upon phylogenetic placement of reads shown in A. The data points are scaled to reflect soil Olsen-P (mg kg^−1^, see Table 1). Model *r*^2^ = 0.650 with dbRDA1 accounting for 87.0% of the fitted and 56.6% of the total variation and dbRDA2 accounting for 13.0% of the fitted and 8.5% of total variation. Partial correlations: dbRDA1 %SOC *r* = −0.093, Ca_ex_*r* = −0.996; dbRDA2 Ca_ex_*r* = −0.093, %SOC = 0.996. The length and direction of each vector indicates the strength and direction of increase, respectively, of the relationship between that variable and the dbRDA axes. The circle is a unit circle (radius = 1.0), the relative size and position of its origin (centre) is arbitrary with respect to the underlying plot. The colours used to differentiate different treatments in B are consistent with A. (For interpretation of the references to colour in this figure legend, the reader is referred to the Web version of this article.)

*Non-specific acid phosphatase genes* – Of the three classes of NSAPs, class B was not found in any substantial numbers (less than 16 reads per metagenome) and so was not analysed further. However, classes A and C were found in significant numbers and responded to soil fertility management. Significant effects were evident for class A gene normalized relative abundance and BWPD_1_ (Fig. 3 and Supplementary Appendix Fig. S3). Class A normalized relative abundance in fertilizer^*+NP*^ soil was significantly greater than in either fertilizer^*-N*^ or fertilizer^*-P*^ soils (smallest difference, *Q* = 7.1, *p* = 0.005): these two treatments were associated with the lowest normalized relative abundance of all treatments. NSAP class A normalized relative abundance was also greater in manure amended soil than fertilizer^*-P*^ soil (*Q* = 4.6, *p* = 0.046). In addition, class A gene BWPD_1_ was also greatest in fertilizer^*+NP*^ soil and significantly greater than ecotype BWPD_1_ in either manure amended or fertilizer^*-N*^ soils (smallest difference, *Q* = 5.4, *p* = 0.022). Fertilizer^*-P*^ soils were associated with intermediate BWPD_1_ and not significantly different from either group of treatments. Edge-PCA (Supplementary Appendix Fig. S7) of phylogenetic placement of metagenome reads (Fig. 7A) separated fertilizer^*+NP*^ soils from the other treatments on the principal axis. Manure amended soil was separated from inorganic fertilizer amended soils on the second axis. No heterogeneity of multivariate dispersion was detected (*pseudo-F* = 1.7, *p*_perm_ = 0.414) but significant differences between treatments based upon NSAP class A phylogeny were detected (*pseudo-F* = 6.6, *p*_perm_ = 6 × 10^−5^). Pair-wise tests indicated that fertilizer^*+NP*^ soil was significantly different from all other treatments (smallest difference, *pseudo-t* = 3.0, *p*_MC_ = 0.0065) but no other comparisons were significant. Consistent with this, dbRDA (Fig. 7B) separated fertilizer^*+NP*^ soil from the other soils on the primary axis. In contrast to the alkaline phosphatases, Ca_ex_ and %SOC were identified as the best combination of edaphic variables to describe the distribution of treatments, accounting for 65% of total variation. Ca_ex_ was again associated with separation of treatments on the primary axis and accounted for 87% of fitted variation, but unlike for the alkaline phosphatases where Olsen-P was associated with the second axis, %SOC separated treatments on the second axis based upon NSAP class A phylogeny (accounting for 13% of fitted variation).

**Fig. 8:**
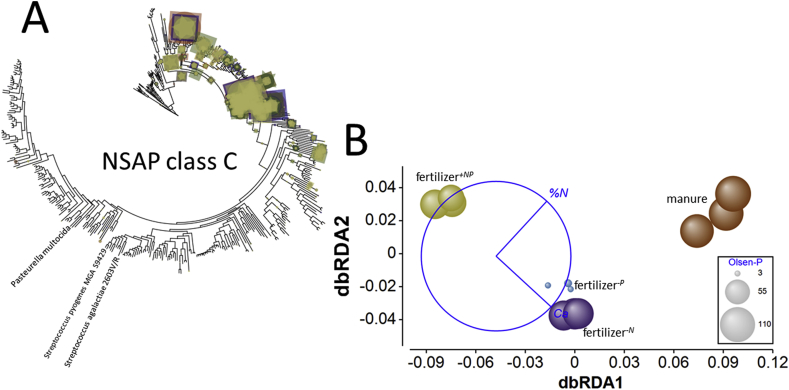
**Effects of soil fertility management upon Class C non-specific acid phosphatase ecotype assemblages in soil.** A – phylogenetic placement of metagenome reads with homology to the nonspecific acid phosphatase class C gene in Broadbalk soils receiving farmyard manure, inorganic fertilizer (NPKMg, fertilizer^*+NP*^), inorganic fertilizer with no nitrogen addition (fertilizer^*-N*^) and inorganic fertilizer with no phosphorus addition (fertilizer^*-P*^). Placement symbol size is scaled to reflect normalized relative abundance across the twelve samples. Different placement shapes – circle, square, star - represent replicates within each treatment. Labels refer to genes described by Gandhi and Chandra (2012). B – Kantorovich-Rubinstein distance-based RDA ordination and partial correlations of exchangeable calcium (Ca) and %N. Kantorovich-Rubinstein distances are based upon phylogenetic placement of reads shown in A. The data points are scaled to reflect soil Olsen-P (mg P kg^−1^, see Table 1). Model *r*^2^ = 0.556 with dbRDA1 accounting for 80.2% of the fitted and 44.6% of the total variation and dbRDA2 accounting for 19.8% of the fitted and 11.0% of total variation. Multiple partial correlations: dbRDA1 %N *r* = 0.678, Ca_ex_*r* = 0.735; dbRDA2 Ca_ex_*r* = −0.678, %N = 0.735. The length and direction of each vector indicates the strength and direction of increase, respectively, of the relationship between that variable and the dbRDA axes. The circle is a unit circle (radius = 1.0), the relative size and position of its origin (centre) is arbitrary with respect to the underlying plot. The colours used to differentiate different treatments in B are consistent with A. (For interpretation of the references to colour in this figure legend, the reader is referred to the Web version of this article.)

For class C genes, the was no significant effect of soil treatment upon gene normalized relative abundance (Fig. 3). However, as with class A genes, BWPD_1_ was greatest in fertilizer^*+NP*^ soil (Supplementary Appendix Fig. S3), significantly greater than in manure amended or fertilizer^*-N*^ soil (smallest difference, *Q* = 4.6, *p* = 0.048). Manure amended soil presented the least diverse assemblage of NSAPc ecotypes of all the soils. No other diversity comparisons were significantly different. Accordingly, edge-PCA (Supplementary Appendix Fig. S8) of phylogenetic placement of metagenome reads (Fig. 8A) separated manure amended from all fertilizer amended soils on the primary axis in a pattern consistent with 16S rRNA gene distribution. No significant heterogeneity of multivariate dispersion was detected (*pseudo-F* = 3.3, *p*_perm_ = 0.070) but a significant treatment effect was determined by PERMANOVA (*pseudo-F* = 4.8, *p*_perm_ = 0.0003). Pair-wise comparison indicated significant differences between all treatments, except fertilizer^*-N*^ – fertilizer^*-P*^ (*pseudo-t* = 1.1, *p*_MC_ = 0.317). Ca_ex_ and %N were identified by dbRDA (Fig. 8B) as the best combination of edaphic variables describing the differences between the treatments, accounting for 57% of total variation. Treatments were separated on the primary axis, largely based upon Ca_ex_ (accounting for 80% of fitted variation) and on the second axis by %N (accounting for 20% of fitted variation), consistent with NSAP class A separation and in contrast to the alkaline phosphatases, separation on the second axis was not dependent upon Olsen-P.

**Fig. 9:**
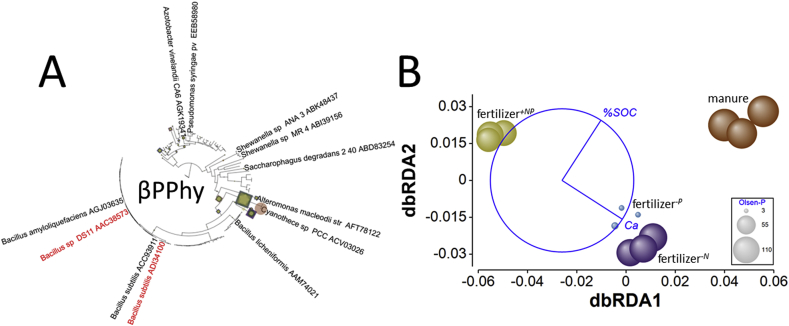
**Effects of soil fertility management upon β-propeller phytase (βPPhy) ecotype assemblages in soil.** A – phylogenetic placement of metagenome reads with homology to the β-propeller phytase gene in Broadbalk soils receiving farmyard manure, inorganic fertilizer (NPKMg, fertilizer^*+NP*^), inorganic fertilizer with no nitrogen addition (fertilizer^*-N*^) and inorganic fertilizer with no phosphorus addition (fertilizer^*-P*^). Placement symbol size is scaled to reflect normalized relative abundance across the twelve samples. Different placement shapes – circle, square, star - represent replicates within each treatment. Black labels refer to genes described by Lim et al. (2007), red labels refer to SWISS-PROT accessions. B – Kantorovich-Rubinstein distance-based RDA ordination and partial correlations of exchangeable calcium (Ca) and %SOC. Kantorovich-Rubinstein distances are based upon phylogenetic placement of reads shown in A. The data points are scaled to reflect soil Olsen-P (mg P kg^−1^, see Table 1). Model *r*^2^ = 0.455 with dbRDA1 accounting for 74.2% of the fitted and 33.7% of the total variation and dbRDA2 accounting for 25.8% of the fitted and 11.7% of total variation. Multiple partial correlations: dbRDA1 %SOC *r* = 0.542, Ca_ex_*r* = 0.840; dbRDA2 Ca_ex_*r* = −0.542, %SOC = 0.840. The length and direction of each vector indicates the strength and direction of increase, respectively, of the relationship between that variable and the dbRDA axes. The circle is a unit circle (radius = 1.0), the relative size and position of its origin (centre) is arbitrary with respect to the underlying plot. The colours used to differentiate different treatments in B are consistent with A. (For interpretation of the references to colour in this figure legend, the reader is referred to the Web version of this article.)

*Myo-inositol hexakisphosphate phosphohydrolase genes* – Of the three phytase genes studied here, βPPhy genes were most abundant, although less abundant than phosphatase genes. The βPPhy was unusual amongst the genes in that it had greatest relative abundance in manure amended and fertilizer^*-P*^ soils (Fig. 3). Normalized relative abundance in these two soils was significantly greater than in either fertilizer^*+NP*^ or fertilizer^*-N*^ soils (smallest difference, *Q* = 20.9, *p* = 1.8 × 10^−6^). The same pattern was observed for BWPD_1_ (Supplementary Appendix Fig. S3), where manure amended and fertilizer^*-P*^ soils were associated with more phylogenetically diverse assemblages than either fertilizer^*+NP*^ or fertilizer^*-N*^ soils. However, only the difference in diversity between manure amended and fertilizer^*+NP*^ soils was significant (*Q* = 5.7, *p* = 0.016). Ordination of the treatments using edge-PCA (Supplementary Appendix Fig. S9) based upon the phylogenetic placement of metagenome reads (Fig. 9A) supported this observation: clusters were not well defined according to treatment, except that the fertilizer^*+NP*^ soil was separated from the other soils on the primary axis. There was no significant heterogeneity of multivariate dispersion (*pseudo-F* = 2.0, *p*_perm_ = 0.229) between treatments, but PERMANOVA identified significant differences in βPPhy KR distance metrics between treatments (*pseudo-F* = 3.1, *p*_perm_ = 0.0013). Only the difference between manure amended and fertilizer^*+NP*^ soil was significant (*pseudo-t* = 2.5, *p*_MC_ = 0.0231). Ca_ex_ and %SOC were identified by dbRDA as the best combination of edaphic factors describing the distribution of treatments and accounting for 45% of total variation. Constrained dbRDA ordination (Fig. 9B) separated fertilizer^*+NP*^ soil from manure amended soil on the primary access according to Ca_ex_ and accounting for 74% of fitted variation, while fertilizer^*+NP*^ and manure amended soils were separated from fertilizer^*-N*^ and fertilizer^*-P*^ soils on the second axis according to %SOC, which was lower in the latter treatments and accounted for 26% of fitted variation. In this respect, βPPhy gene phylogeny was consistent with NSAP genes, which showed no response to Olsen-P differences between the treatments.

For the acidic phytases (CPhy and HAPhy) there were only limited differences between treatments (Fig. 3 and Supplementary Appendix Fig. S3). In both cases, there was no significant effect of soil management upon gene BWPD_1_ and normalized relative abundance was significantly greater in fertilizer^*+NP*^ soil than either fertilizer^*-P*^ or fertilizer^*-N*^ soils (CPhy smallest difference, *Q* = 5.1, *p* = 0.029; HAPhy smallest difference, *Q* = 4.8, *p* = 0.036). CPhy was associated with a significant heterogeneity of multivariate dispersion (*pseudo-F* = 8.5, *p*_perm_ = 0.0002) and this is evident from edge PCA ordination (Supplementary Appendix Fig. S10) with much greater dispersion associated with fertilized and reduced dispersion associated with manure amended soils. However, there was no significant treatment effect upon CPhy phylogeny (*pseudo-F* = 2.2, *p*_perm_ = 0.071). For HAPhy, there was neither significant heterogeneity of multivariate dispersion (*pseudo-F* = 2.0, *p*_perm_ = 0.334) or a significant treatment effect upon phylogeny (*pseudo-F* = 0.6, *p*_perm_ = 0.795).

## 4 Discussion

There is evidence that soil management exerts a direct influence upon soil microbial communities. In some instances, community and functional changes are observed in agricultural soils and between different agricultural practices. For example, soils managed by the addition of animal-derived or green manures may contain more abundant and active microbial communities than inorganic fertilizer amended soils (Lori et al., 2017). These communities are often also more diverse (Zhen et al., 2014; Francioli et al., 2016; Li et al., 2017; Li et al., 2017). However, it is typically not a general loss of diversity that is responsible for a loss of function, but loss of specific species or functional groups within the wider assemblage (Bier et al., 2015). On the Broadbalk winter wheat experiment, addition of farmyard manure or inorganic fertilizer for over 170 years has not induced significant changes to 16S rRNA phylogenetic diversity. The unprecedented timespan of the Broadbalk experiment may explain the lack of differences in phylogenetic diversity, in the sense that communities subject to the different treatments have effectively had time reach stable end-point structures, less likely in shorter experiments. Although phylogenetic diversity was unchanged between treatments, distinct 16S rRNA assemblages were detected under the different treatments, directly influenced by the nature of the nutrient amendment although the specific response of these assemblages differed from those observed on the Swiss DOK long-term experiment (Hartmann et al., 2015) where soil organic carbon, total nitrogen and pH were the strongest predictors of community structure. On Broadbalk, organisms such as *Pirellula staleyi*, *Woeseia oceani* and *Steriodobacter denitrificans* were most abundant in manure amended soils. Rather unexpectedly, the obligate nematode endosymbiont (Vandekerckhove et al., 2002) *Ca.* Xiphinematobacter was most abundant in, and characteristic of fertilized soil: dagger nematodes (*Xiphinema* spp.) are ectoparasites of various plant crop species, including cereals, and potentially act as vectors for several economically important plant viruses (McFarlane et al., 2002).

Comparison of estimates of mean phylogenetic diversity indicate that assemblages of phosphohydrolase gene ecotypes are more sensitive to fertility management than the 16S rRNA-conditional microbial community and respond in a fundamentally different manner. Despite sharing a common function, each phosphohydrolase gene has a distinct relative abundance and phylogenetic diversity profile – and response to management - within the soil communities studied here. The alkaline phosphatase *phoD* was the most abundant and phylogenetically diverse phosphohydrolase gene. This gene is the most abundant alkaline phosphatase in both marine and soil systems (Luo et al., 2009; Neal et al., 2017), but *phoD* is also more abundant than any non-specific acid phosphatase or phytase genes. A group of genes appear to share a similar relative abundance, but different phylogenetic diversity: these include a second alkaline phosphatase, *phoX*, class A and C NSAPs and βPPhy genes. Of these, *phoX* is the most abundant and phylogenetically diverse and βPPhy the least. The remaining genes are all of low relative abundance and phylogenetic diversity, suggesting that alkaline phosphatase *phoA*, class B NSAP and the acidic phytase CPhy and HAPhy genes do not contribute significantly to hydrolysis of P_org_ in soils, consistent with their presence largely in enteric or pathogenic bacteria (Neal et al., 2017,Neal et al., 2018). Based upon the substrate specificity of the different enzyme groups (Rossolini et al., 1998; Luo et al., 2009) the observed relative abundance of phosphohydrolase genes in soil suggests that phosphomono- and phosphodiesters are principal sources of P for soil microbes, and that phytate is not a major source.

Our aim in this study was to test hypotheses established from amplicon sequencing approaches regarding the relative abundance and diversity of phosphohydrolase genes, using metagenomic approaches. In common with amplicon approaches, our approach identifies partial gene sequences upon the basis of homology to a set of reference genes and so represents potential rather than actual activity. Our assumption is that over the 170-year history of the experiment, communities will evolve within the background of the prevailing chemistry and the resulting abundance of genes will reflect their relative utility under the different environments. We made use of soils with a history of fertility management either with farmyard manure or various combinations of inorganic fertilizer. It is generally accepted that orthophosphate bioavailability directly controls the enzymatic activity and gene abundance of phosphohydrolases: we used Olsen-P estimates as a measure of orthophosphate bioavailability. We would therefore expect soils associated with a long history of low Olsen-P concentrations because of fertility management – the fertilizer^*-P*^ treatment in this case - to be associated with relatively high abundance of various P_org_-related genes. Conversely, treatments associated with persistently high Olsen-P – manure amended and fertilizer^*-N*^ treatments, particularly – should be associated with a relatively low gene abundance. Our results indicated that for both gene relative abundances and phylogenetic diversity (BWPD_1_), differences between manure (Olsen-P, 98 mg P kg^−1^) and fertilizer^*-P*^ (Olsen-P, 3 mg P kg^−1^) amended soils were minimal, indicating no direct relationship between orthophosphate availability and gene relative abundance. Gene relative abundance in fertilizer^*+NP*^ soil was quite distinct from other treatments. Relative abundance of *phoD*, *phoX* and βPPhy genes were lowest in fertilizer^*+NP*^ soil, despite it having comparable Olsen-P concentrations to manure amended and fertilizer^*-N*^ soils; normalized relative abundance of *phoA*, NSAP class A and CPhy and HAPhy genes were highest in fertilizer^*+NP*^ soil, again in a manner inconsistent with bioavailable orthophosphate being an important determining factor. Thus, consistent and significant differences were observed in gene relative abundance between treatments that were inconsistent with bioavailable orthophosphate being the dominant determining factor.

In addition to normalized relative abundance and phylogenetic diversity, we also assessed the phylogenetic differences between gene assemblages in the different treatments using Kantorovich-Rubinstein (KR) distance metrics, derived from phylogenetic placement of homologous sequences identified in the metagenomic datasets. Distance-based redundancy analysis (dbRDA) using KR metrics supported relative abundance- and phylogenetic diversity-based evidence for a lack of strong influence of Olsen-P. An influence of Olsen-P upon alkaline phosphatase gene phylogeny was suggested by dbRDA (*phoD*, *phoX* and *phoA*, see Fig. 4, Fig. 5, Fig. 6B) which was not observed for NSAP or phytase genes, however this effect was consistently less than the effect of Ca_ex_. For all genes, Ca_ex_ was the dominant edaphic factor influencing KR distance metrics. For the alkaline phosphatase genes, there was a consistent pattern regarding ordination of the KR distance metrics in that assemblages of the respective genes in manure and fertilizer^*-N*^ soils were phylogenetically more similar than for the other treatments, despite the soils having quite distinct N inputs and content – 0.28% N for manured soil, but 0.095% N for fertilizer^*-N*^ soil. There is some evidence that N-addition to soils, either as inorganic fertilizer or animal manures, can reduce the abundance and diversity of *phoD* genes in soil (Chen et al., 2019) which is primarily a response to the effects of N-additions upon pH. In our soils, where pH is maintained by liming, we observed no consistent association between N-addition, whether from inorganic or organic sources or with associated P or not. The effect of Ca_ex_ was more pronounced than any effect associated with N.

The observation that Olsen-P was less strongly associated with the distribution of phosphohydrolase genes than Ca_ex_, despite a broad range across the experimental treatments was unexpected and contrary to previous studies of the effects of edaphic factors upon gene diversity (Ragot et al., 2017), although not without precedent (Fraser et al., 2015). On Broadbalk, PHO gene ecotype assemblages and relative abundance were typically very similar in fertilizer^*-N*^ and fertilizer^*-P*^ soils, despite significantly different Olsen-P estimates of 88 and 3 mg kg^−1^ respectively. Observations of the effect of P chemistry upon gene diversity were apparent from a study of soils from a wide variety of geographical locations, and with a wide variety of P-chemistry and availability (Ragot et al., 2017), whereas this study and that of soil from the Glenlea Research Station, Manitoba (Fraser et al., 2015) are local studies, comparing soils of relatively similar chemistry *etc*. It is possible that different edaphic factors influence gene distribution and diversity at different scales, especially since pH is typically manipulated in arable soils. In the Broadbalk soils, Olsen-P appeared to exert a minor influence only upon the alkaline phosphatases. The acid phosphatases and phytases appeared not to respond to Olsen-P in the soils.

For many of the PHO genes studied here, there was a striking association between ecotype relative abundance and phylogenetic assemblage with Ca_ex_ identified by dbRDA, and where this was the case Ca_ex_ accounted for a far greater proportion of fitted variability than any other edaphic factor. The soil is slightly calcareous, but Ca is also derived from liming of soil to maintain optimal pH for crop yield. Between 1954 and 2015, total chalk (CaCO_3_) applications of 39.1 t ha^−1^ to fertilizer^*+NP*^, 25.1 t ha^−1^ to fertilizer^*-P*^ and 14.7 t ha^−1^ to each of the other two treatments were made. Precipitation of phosphorus in various calcium phases (di- and octacalcium phosphate and hydroxyapatite) is typically the predominant mechanism controlling P bioavailability in soils with a high reservoir of exchangeable cations (von Wandruszka, 2006). It is possible therefore that gene abundance indirectly reflects the bioavailability of orthophosphate modulated by Ca_ex_. In this study there is no clear relationship between Olsen-P and Ca_ex_ (Table 1): soils associated with over 6 g kg^−1^ Ca_ex_ are simultaneously associated with extremes of Olsen-P (97 and 3 mg P kg^−1^). Strong sorption of orthophosphate to mineral surfaces in Broadbalk soils is only important below approximately 60 mg P kg^−1^, *i.e*. in fertilizer^−*P*^ soil. Above this threshold sorption energy is reduced, promoting orthophosphate mobility and bioavailability (Heckrath et al., 1995). For most genes, dbRDA indicated that assemblages identified in fertilizer^*+NP*^ soil were distinctly different from those in the other soils. This soil was associated with significantly lower Ca_ex_ than the other soils. In some calcareous soils, the organic proportion of total P is positively correlated with Ca content (Harrison, 1987), but the response of gene relative abundance and phylogeny across the experiment does not suggest that this is the cause of the differences we observe. Alternatively, PhoD and PhoX enzymes both require Fe^3+^ and Ca^2+^ as cofactors (Rodriguez et al., 2014; Yong et al., 2014) and βPPhy also requires Ca^2+^ (Mullaney and Ullah, 2003). The other proteins all require different co-factors; Zn^2+^ and Mg^2+^ for PhoA (Torraini, 1990), VO_4_^3−^ for the NSAPs (Littelchild et al., 2002). The acid phytases CPhy and HAPhy have no known requirement for metal cofactors. Genes coding for the three Ca^2+^-dependant proteins show either significantly reduced relative abundance (*phoX*, βPPhy) or significantly increased phylogenetic diversity (*phoD*) compared to the other soils. Genes coding for Ca^2+^-independent proteins show significantly increased relative abundance in fertilizer^*+NP*^ soil (*phoA*, NSAP class A, CPhy and HAPhy), perhaps in response reduced effectiveness of PhoD and PhoX. If this is the case, it is evident that some *phoD* ecotypes appear to code for enzymes which are more efficient at reduced Ca^2+^ availability since the phylogenetic diversity in fertilized soil is significantly increased in this treatment. This may also explain why *phoD* is the most abundant PHO gene globally. In contrast, the general distribution of *phoX* and βPPhy ecotypes is much more restricted, suggesting greater sensitivity to Ca availability. Whatever the cause, the result is that function - in this case hydrolysis of P_org_ compounds to release orthophosphate – is maintained within the background of changing 16S rRNA-contingent community structure and cofactor availability. The genes (and thus proteins) responsible for this function are selected based upon environmental fitness, probably to chemical edaphic factors, but not P-availability.

Our metagenomic studies of genes coding for phosphohydrolase genes in soil did not support the hypothesis that gene abundance reflects the bioavailability of orthophosphate in soil. One possible explanation for this difference is that shotgun metagenomic approaches reveal a far greater biodiversity than primer-based studies of the various genes, but it is also worth noting that like primer-based studies which largely address gene abundance in extracted DNA, shotgun metagenomic approaches can only reveal the potential for enzyme expression and activity. A key assumption for all such studies is that observed gene abundance (and inferred potential) reflects the prevailing soil environment, such as bioavailable P. However, in the majority of studies environmental parameters are measured over a limited temporal span and often only once, providing limited insight into the range of conditions experienced by microbial communities within soil. Given the longevity of the Broadbalk winter wheat experiment and the availability of Olsen-P estimates spanning over 170 years, we can be sure that communities in manure amended, fertilizer^*+NP*^ and fertilizer^*-N*^ soils have evolved within, and reflect, a background of continually increasing Olsen P (notwithstanding reductions since 2000), in stark contrast to the community in fertilizer^*-P*^ soil. It is likely therefore that long-term studies such as this provide a more accurate reflection of genetic responses to nutrient availability. Our study suggests the following hypothesis: bioavailability of enzyme cofactors (Ca_ex_ in the case of *phoD*, *phoX* and βPPhy studied here) influence the relative abundance of genes in soil microbial communities; in the absence of important cofactors, genes coding for alternative enzyme families not requiring the limiting cofactor (for example non-specific acid phosphatases which require vanadate) become more abundant. In this way, the general function – that of hydrolysing organic P compounds to release orthophosphate – is maintained in the community. If this hypothesis is supported by future testing, it suggests that Ca_ex_ is an important edaphic factor to consider for effective and efficient management of organic phosphorus in soils.
