# Supplementary material for: Calcium exerts a strong influence upon phosphohydrolase gene abundance and phylogenetic diversity in soil
Source: Soil Biol Biochem. 2019 Dec;139:107613. doi: 10.1016/j.soilbio.2019.107613 (PMC6919939; doi:10.1016/j.soilbio.2019.107613)
Supplement: Multimedia component 1 [file mmc1.docx]

Calcium Exerts a Strong Influence upon Phosphohydrolase Gene Abundance and Phylogenetic Diversity in Soil.

# Andrew L. Neal^1†^ and Margaret J. Glendining^2^

^1^Department of Sustainable Agriculture Sciences and ^2^Computational and Analytical Sciences, Rothamsted Research, Harpenden, Hertfordshire, UK.

†Corresponding author: andy.neal@rothamsted.ac.uk; Rothamsted Research, Harpenden, Hertfordshire, AL5 2JQ. UK. +44 (0)1582 763133

Supplementary Appendix


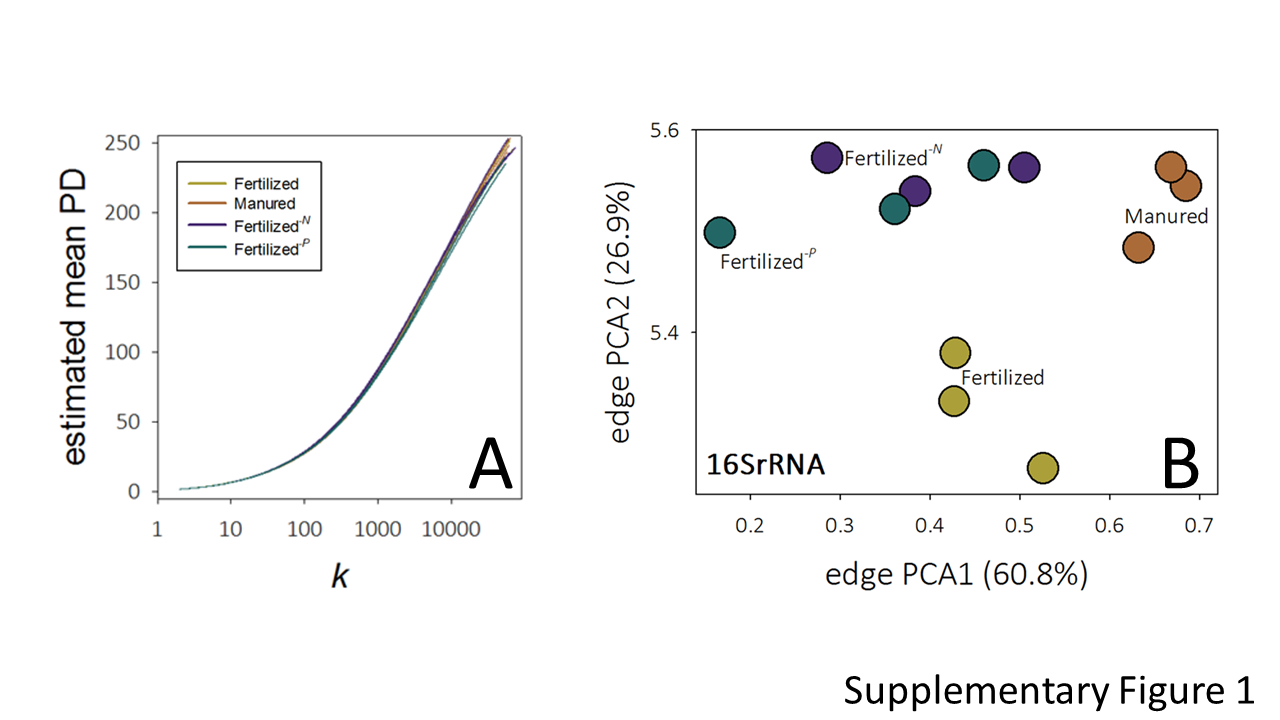


**SUPPLEMENTARY FIGURE 1**. **Effects of soil fertility management upon the microbial assemblages in soil according to 16S rRNA homology**. **A** – estimated mean unrooted phylogenetic diversity (PD – expressed as the sum of branch lengths occupied on the gene phylogenetic tree) of the 16S rRNA gene assemblages at increasing rarefaction size (*k*) identified in soils receiving farmyard manure, inorganic fertilizer (NPKMg, fertilizer), inorganic fertilizer with no nitrogen addition (fertilizer*^-N^*) and inorganic fertilizer with no phosphorus addition (fertilizer*^-P^*). **B** – edge-PCA ordination, based upon the abundance-weighted placements in Figure 2A, of the gene assemblages present in each soil.


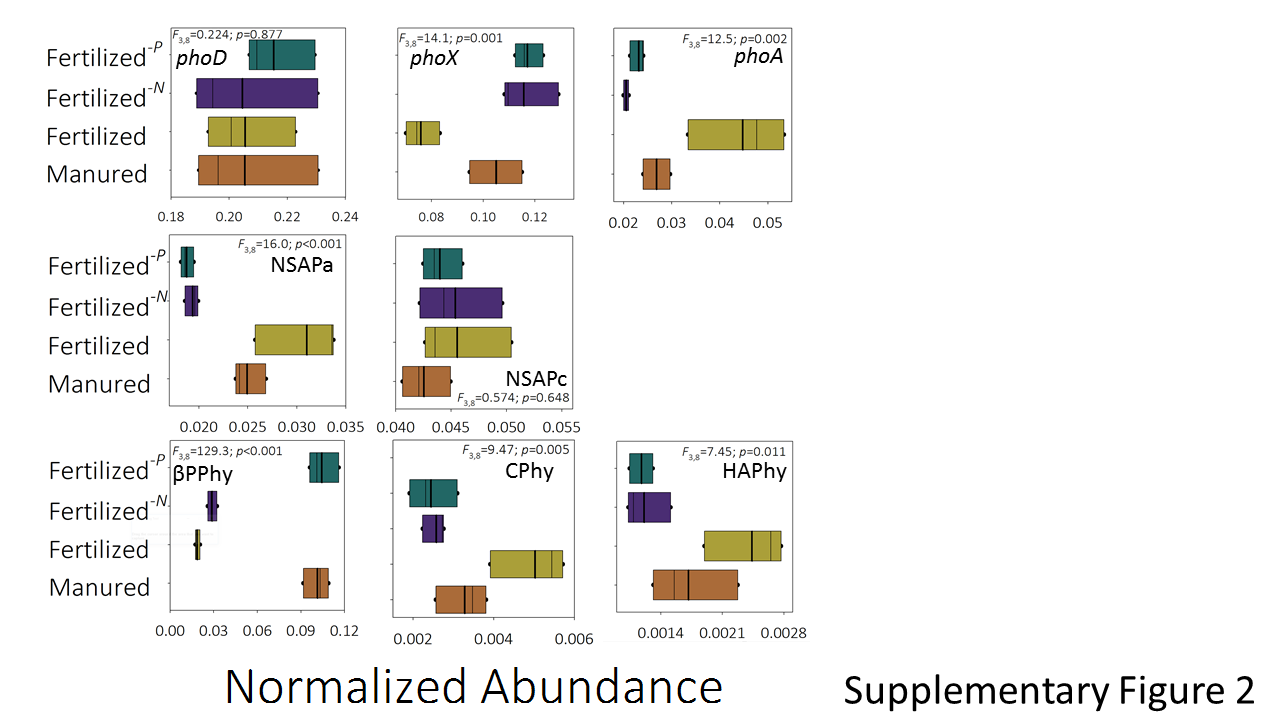


**SUPPLEMENTARY FIGURE 2**. **Abundance of Phosphohydrolase Genes in Broadbalk Soil**. **L**ength-normalized abundance (relative to the 16S rRNA gene, see Materials and Methods section for calculation description) of gene ecotypes in soils receiving farmyard manure, inorganic fertilizer (NPKMg, fertilized), inorganic fertilizer with no nitrogen addition (fertilized*^-N^*) and inorganic fertilizer with no phosphorus addition (fertilized*^-P^*).


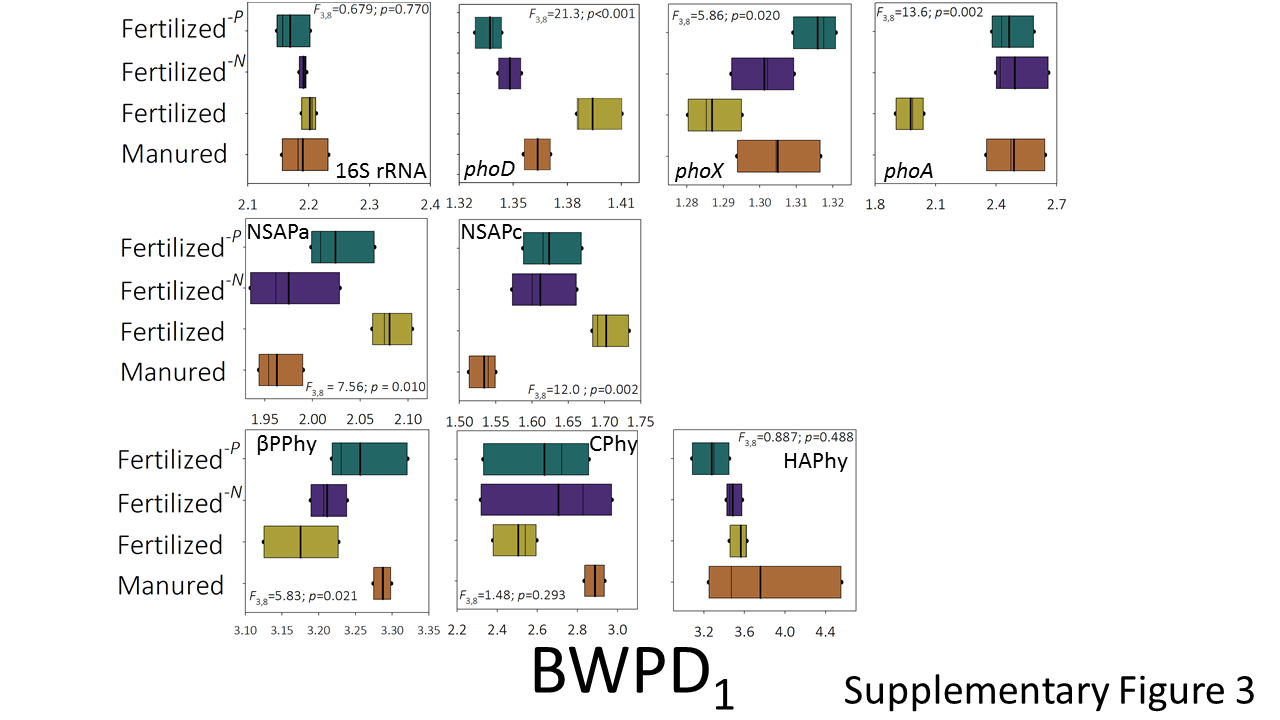


**SUPPLEMENTARY FIGURE 3**. **Balance-weighted phylogenetic diversity (BWPD_1_) of gene ecotypes in soils** receiving farmyard manure, inorganic fertilizer (NPKMg, fertilized), inorganic fertilizer with no nitrogen addition (fertilized*^-N^*) and inorganic fertilizer with no phosphorus addition (fertilized*^-P^*).


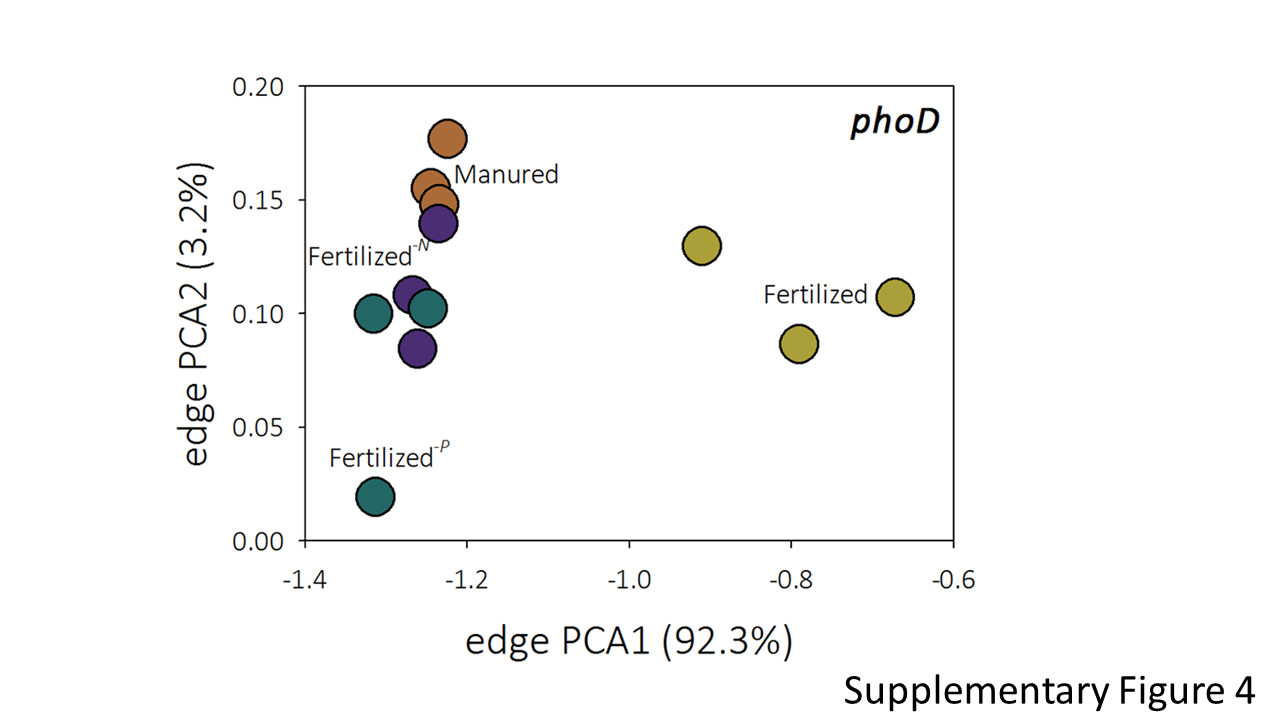


**SUPPLEMENTARY FIGURE 4**. **Edge-PCA of the Alkaline Phosphatase *phoD* Gene Ecotypes in Broadbalk Soils.** The ordination is based upon the abundance-weighted *phoD* placements in Figure 4A of gene assemblages present in soils receiving farmyard manure, inorganic fertilizer (NPKMg, fertilized), inorganic fertilizer with no nitrogen addition (fertilized*^-N^*) and inorganic fertilizer with no phosphorus addition (fertilized*^-P^*).


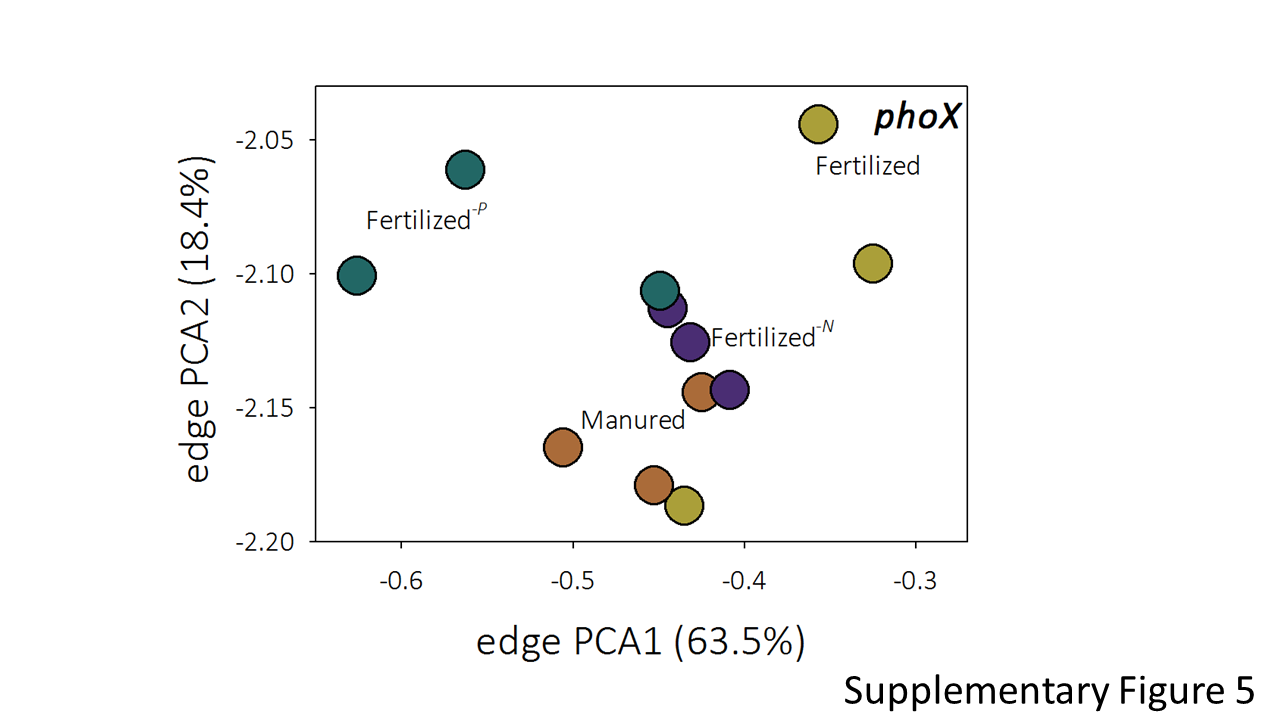


**SUPPLEMENTARY FIGURE 5**. **Edge-PCA of the Alkaline Phosphatase *phoX* Gene Ecotypes in Broadbalk Soils.** The ordination is based upon the abundance-weighted *phoX* placements in Figure 5A of gene assemblages present in soils receiving farmyard manure, inorganic fertilizer (NPKMg, fertilized), inorganic fertilizer with no nitrogen addition (fertilized*^-N^*) and inorganic fertilizer with no phosphorus addition (fertilized*^-P^*).


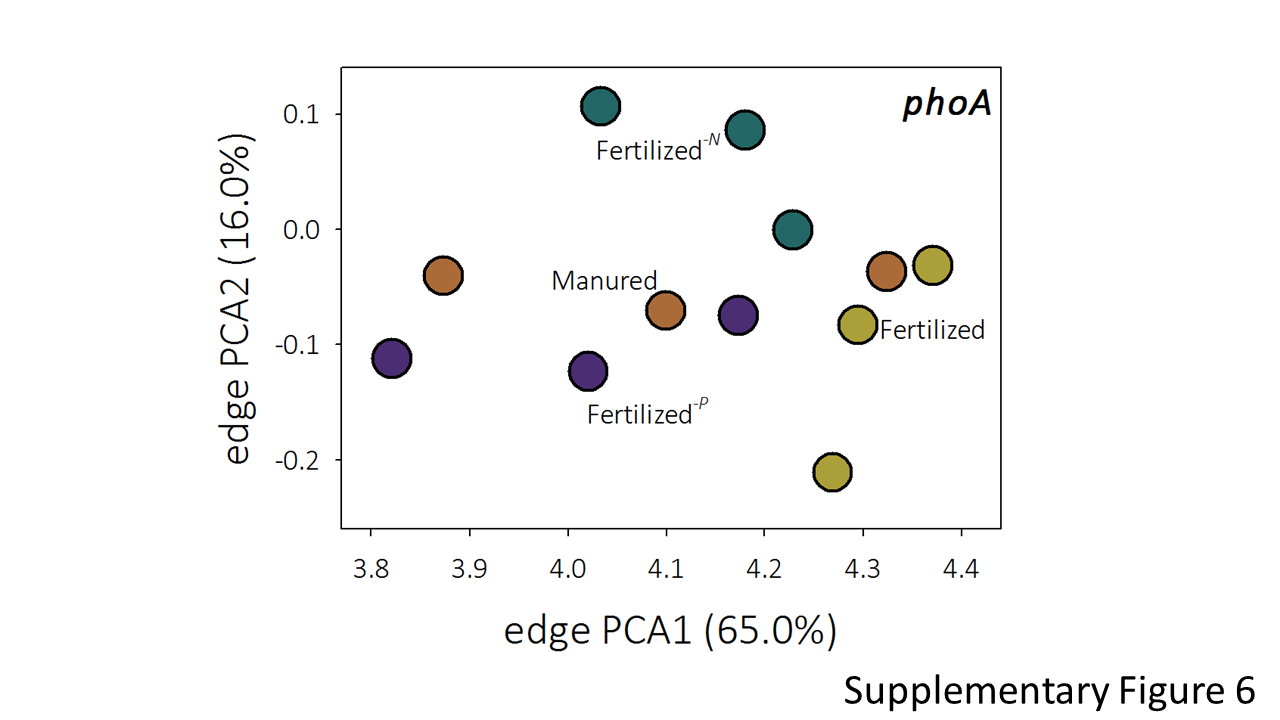


**SUPPLEMENTARY FIGURE 6**. **Edge-PCA of the Alkaline Phosphatase *phoA* Gene Ecotypes in Broadbalk Soils.** The ordination is based upon the abundance-weighted *phoA* placements in Figure 6A of gene assemblages present in soils receiving farmyard manure, inorganic fertilizer (NPKMg, fertilized), inorganic fertilizer with no nitrogen addition (fertilized*^-N^*) and inorganic fertilizer with no phosphorus addition (fertilized*^-P^*).


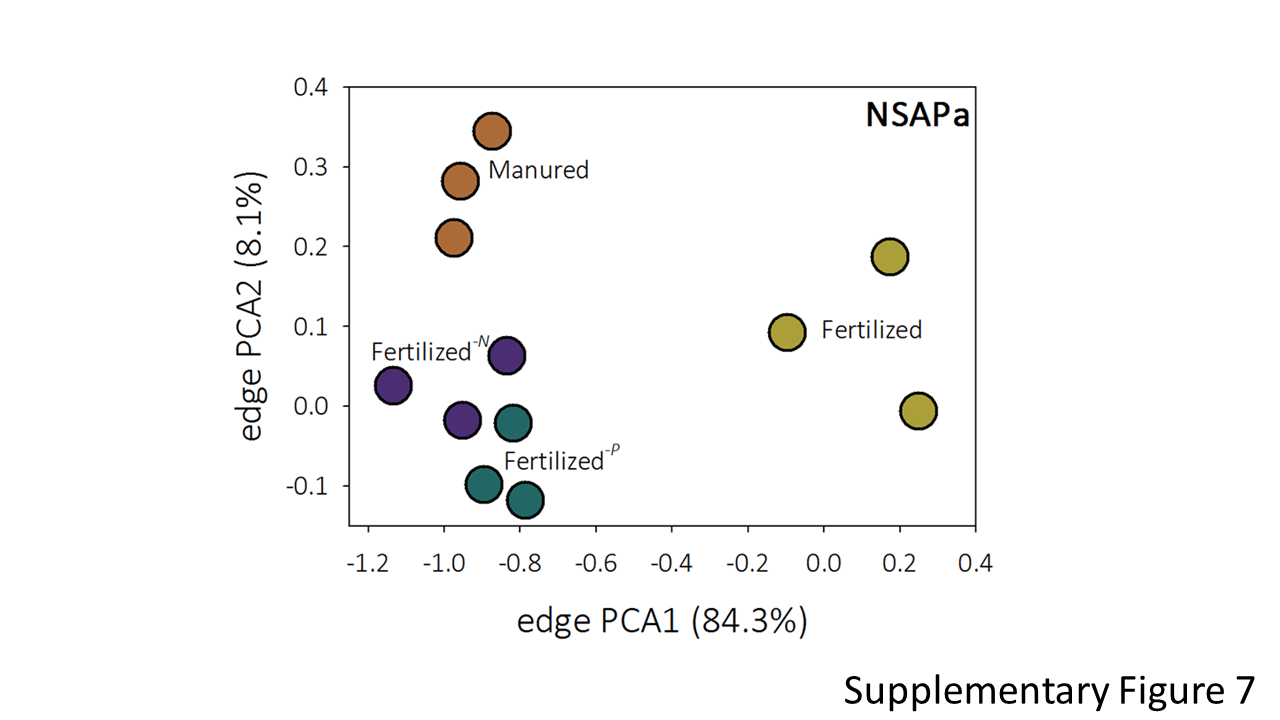


**SUPPLEMENTARY FIGURE 7**. **Edge-PCA of the Non-Specific Acid Phosphatase Class A Gene Ecotypes in Broadbalk Soils.** The ordination is based upon the abundance-weighted NSAPa placements in Figure 7A of gene assemblages present in soils receiving farmyard manure, inorganic fertilizer (NPKMg, fertilized), inorganic fertilizer with no nitrogen addition (fertilized*^-N^*) and inorganic fertilizer with no phosphorus addition (fertilized*^-P^*).


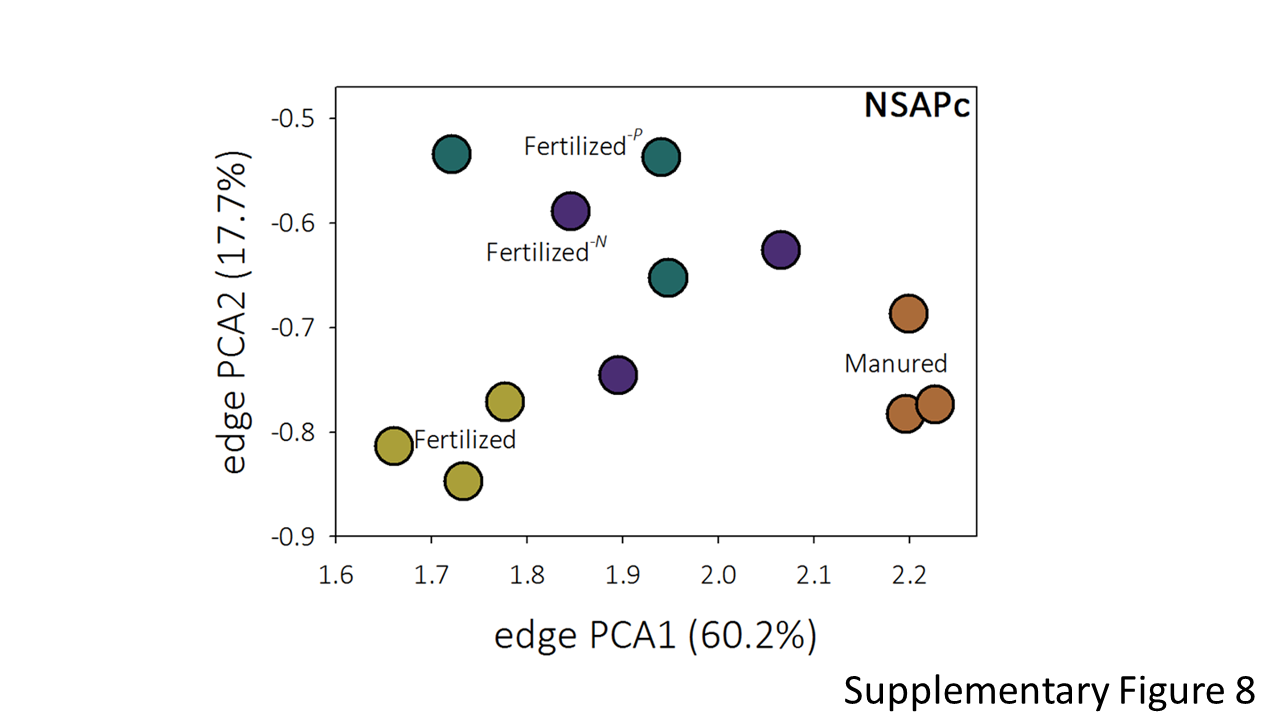


**SUPPLEMENTARY FIGURE 8**. **Edge-PCA of the Non-Specific Acid Phosphatase Class C Gene Ecotypes in Broadbalk Soils.** The ordination is based upon the abundance-weighted NSAPc placements in Figure 7A of gene assemblages present in soils receiving farmyard manure, inorganic fertilizer (NPKMg, fertilized), inorganic fertilizer with no nitrogen addition (fertilized*^-N^*) and inorganic fertilizer with no phosphorus addition (fertilized*^-P^*).


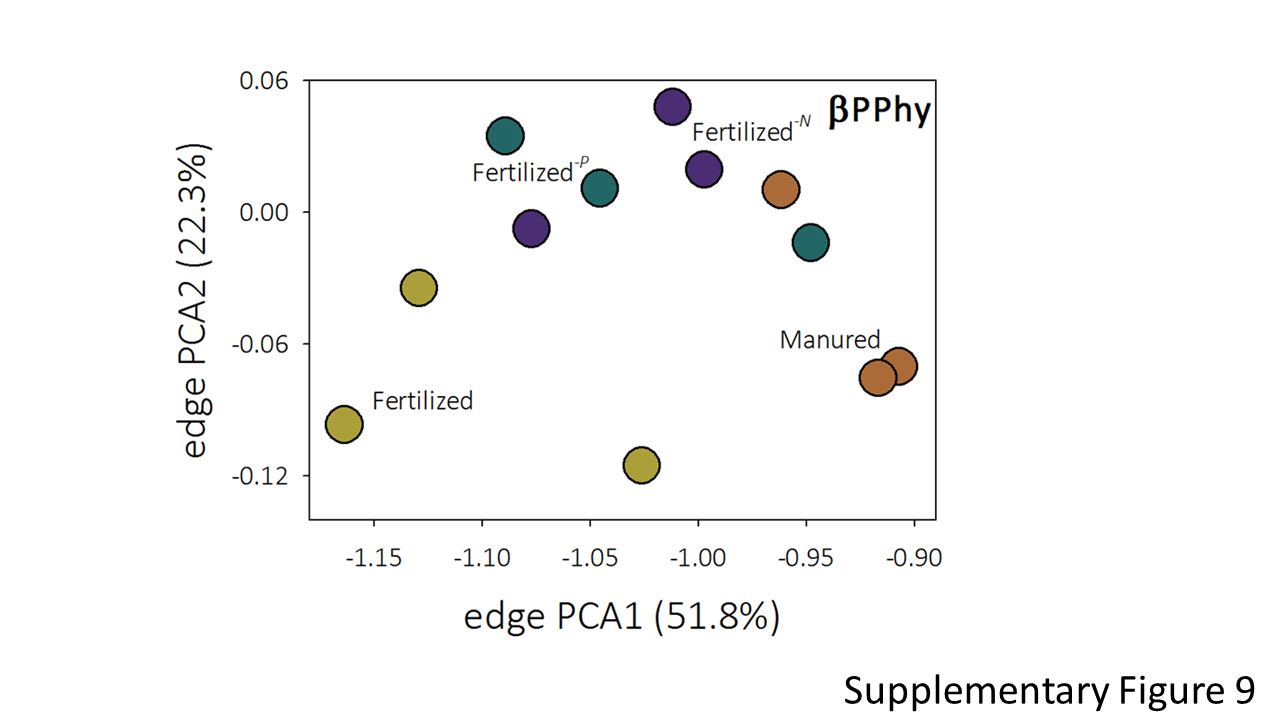


**SUPPLEMENTARY FIGURE 9**. **Edge-PCA of the β-Propeller Phytase Gene Ecotypes in Broadbalk Soils.** The ordination is based upon the abundance-weighted βPPhy placements in Figure 9A of gene assemblages present in soils receiving farmyard manure, inorganic fertilizer (NPKMg, fertilized), inorganic fertilizer with no nitrogen addition (fertilized*^-N^*) and inorganic fertilizer with no phosphorus addition (fertilized*^-P^*).


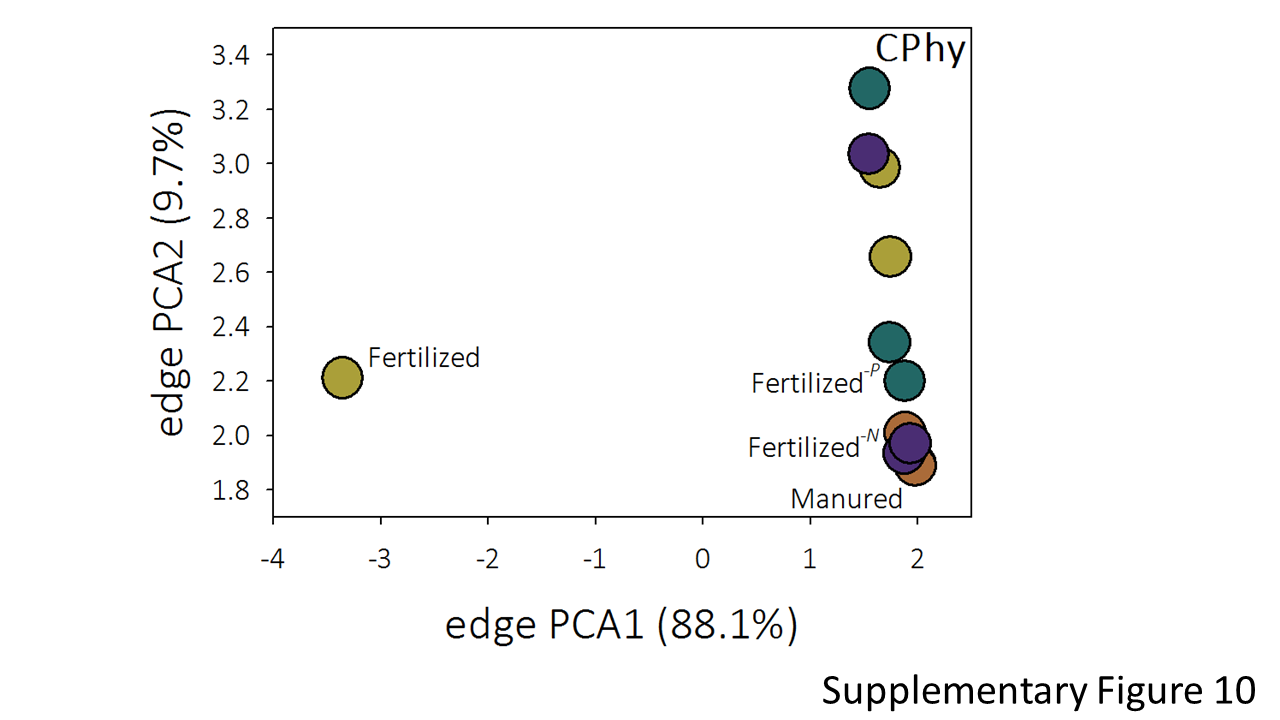


**SUPPLEMENTARY FIGURE 10**. **Edge-PCA of the Cysteine Phytase Gene Ecotypes in Broadbalk Soils.** The ordination is based upon the abundance-weighted CPhy placements of gene assemblages present in soils receiving farmyard manure, inorganic fertilizer (NPKMg, fertilized), inorganic fertilizer with no nitrogen addition (fertilized*^-N^*) and inorganic fertilizer with no phosphorus addition (fertilized*^-P^*).
