# Supplementary material for: Calcium exerts a strong influence upon phosphohydrolase gene abundance and phylogenetic diversity in soil
Source: Soil Biol Biochem. 2019 Dec;139:107613. doi: 10.1016/j.soilbio.2019.107613 (PMC6919939; doi:10.1016/j.soilbio.2019.107613)
Supplement: Multimedia component 2 [file mmc2.docx]

|  | raw reads | useful reads | error rate / % | Phred^a^ *Q*_20_^b^ / % | Phred *Q*_30_^c^ / % |
| --- | --- | --- | --- | --- | --- |
| manure 1 | 430,626,730 | 429,251,644 | 0.0300 | 96.28 | 91.38 |
| manure 2 | 409,547,052 | 408,618,063 | 0.0300 | 96.38 | 91.48 |
| manure 3 | 387,923,009 | 387,232,849 | 0.0300 | 96.70 | 91.98 |
| fertilizer*^+NP^* 1 | 464,621,933 | 390,099,234 | 0.0275 | 96.77 | 92.22 |
| fertilizer*^+NP^* 2 | 413,959,667 | 413,357,210 | 0.0300 | 95.93 | 90.63 |
| fertilizer*^+NP^* 3 | 399,943,440 | 399,258,646 | 0.0300 | 96.67 | 91.95 |
| fertilizer*^-N^* 1 | 390,652,145 | 390,099,234 | 0.0275 | 96.77 | 92.22 |
| fertilizer*^-N^* 2 | 461,939,542 | 460,840,411 | 0.0300 | 96.87 | 93.09 |
| fertilizer*^-N^* 3 | 461,166,668 | 459,875,809 | 0.0300 | 96.32 | 92.14 |
| fertilizer*^-P^* 1 | 367,015,670 | 366,640,102 | 0.0425 | 94.86 | 87.73 |
| fertilizer*^-P^* 2 | 398,797,245 | 398,293,503 | 0.0450 | 94.79 | 87.65 |
| fertilizer*^-P^* 3 | 390,704,624 | 390,095,206 | 0.0425 | 93.80 | 87.06 |
| mean | **414,741,477** | **413,924,080** | **3.3x10^-2^** | **96.05** | **90.85** |
| standard deviation | **31,330,495** | **31,063,801** | **6.0x10^-3^** | **0.987** | **2.047** |

^a^ – Phred scores (*Q*) are logarithmically related to the base calling error probability (*P*), thus

*Q* = -10log_10_*P*

^b^ – 1% probability of incorrect base call

^c^ – 0.1% probability of incorrect base call

**Supplementary Table I.** Summary of total raw reads and useful reads, and quality statistics associated with the metagenome datasets used in this study.
