# Supplementary material for: Calcium exerts a strong influence upon phosphohydrolase gene abundance and phylogenetic diversity in soil
Source: Soil Biol Biochem. 2019 Dec;139:107613. doi: 10.1016/j.soilbio.2019.107613 (PMC6919939; doi:10.1016/j.soilbio.2019.107613)
Supplement: Multimedia component 3 [file mmc3.docx]

|  | *gyrB* | *recA* | *atpD* | Length-normalized estimate of number of “genomes”^a^ | *phoD* | *phoD* relative abundance^b^ | *phoX* | *phoX* relative abundance^b^ | *phoA* | *phoX* relative abundance^b^ |
| --- | --- | --- | --- | --- | --- | --- | --- | --- | --- | --- |
| manure 1 | 78,747 | 28,545 | 43,306 | 33,257 | 19,465 | 58.5 | 8,742 | 26.3 | 1,566 | 4.7 |
| manure 2 | 76,924 | 28,458 | 42,396 | 32,704 | 19,645 | 60.1 | 8,682 | 26.5 | 1,566 | 4.8 |
| manure 3 | 69,255 | 25,710 | 39,646 | 29,885 | 18,129 | 60.7 | 7,638 | 25.6 | 1,358 | 4.5 |
| fertilizer*^+NP^* 1 | 84,488 | 33,045 | 48,114 | 36,948 | 20,960 | 56.7 | 6,428 | 17.4 | 2,139 | 5.8 |
| fertilizer*^+NP^* 2 | 74,977 | 28,753 | 41,246 | 32,193 | 18,498 | 57.5 | 6,222 | 19.3 | 2,790 | 8.7 |
| fertilizer*^+NP^* 3 | 73,643 | 29,173 | 42,115 | 32,378 | 19,268 | 59.5 | 5,953 | 18.4 | 2,670 | 8.2 |
| fertilizer*^-N^* 1 | 71,344 | 27,356 | 39,583 | 30,725 | 17,672 | 57.5 | 8,539 | 27.8 | 1,090 | 3.5 |
| fertilizer*^-N^* 2 | 83,660 | 32,731 | 47,475 | 36,542 | 21,444 | 58.7 | 10,541 | 28.8 | 1,395 | 3.8 |
| fertilizer*^-N^* 3 | 80,268 | 31,291 | 46,786 | 35,367 | 22,050 | 62.3 | 10,571 | 29.9 | 1,207 | 3.4 |
| fertilizer*^-P^* 1 | 65,081 | 25,106 | 36,287 | 28,128 | 16,356 | 58.1 | 7,847 | 27.9 | 1,138 | 4.0 |
| fertilizer*^-P^* 2 | 70,529 | 27,609 | 40,520 | 30,950 | 19,294 | 62.3 | 8,861 | 28.6 | 1,214 | 3.9 |
| fertilizer*^-P^* 3 | 66,236 | 25,514 | 37,151 | 28,676 | 17,123 | 59.7 | 7,868 | 27.4 | 1,046 | 3.6 |

|  | NSAP class A | NSAP class A relative abundance^b^ | NSAP class C | NSAP class C relative abundance^b^ | βPPhy | βPPhy relative abundance^b^ | CPhy | CPhy relative abundance^b^ | HAPhy | HAPhy relative abundance^b^ |
| --- | --- | --- | --- | --- | --- | --- | --- | --- | --- | --- |
| manure 1 | 780 | 2.3 | 1,444 | 4.3 | 7,698 | 23.2 | 140 | 0.42 | 68 | 0.20 |
| manure 2 | 800 | 2.4 | 1,402 | 4.3 | 7,378 | 22.6 | 94 | 0.29 | 73 | 0.22 |
| manure 3 | 768 | 2.6 | 1,352 | 4.5 | 6,630 | 22.2 | 149 | 0.50 | 114 | 0.38 |
| fertilizer*^+NP^* 1 | 930 | 2.5 | 1,646 | 4.5 | 1,454 | 3.9 | 174 | 0.47 | 108 | 0.29 |
| fertilizer*^+NP^* 2 | 1,109 | 3.4 | 1,474 | 4.6 | 1,317 | 4.1 | 232 | 0.72 | 144 | 0.45 |
| fertilizer*^+NP^* 3 | 953 | 2.9 | 1,491 | 4.6 | 1,319 | 4.1 | 189 | 0.58 | 118 | 0.36 |
| fertilizer*^-N^* 1 | 572 | 1.9 | 1,427 | 4.6 | 1,976 | 6.4 | 103 | 0.34 | 50 | 0.16 |
| fertilizer*^-N^* 2 | 765 | 2.1 | 1,696 | 4.6 | 2,261 | 6.2 | 130 | 0.36 | 66 | 0.18 |
| fertilizer*^-N^* 3 | 633 | 1.8 | 1,679 | 4.7 | 2,360 | 6.7 | 89 | 0.25 | 77 | 0.22 |
| fertilizer*^-P^* 1 | 497 | 1.8 | 1,216 | 4.3 | 5,842 | 20.8 | 76 | 0.27 | 44 | 0.16 |
| fertilizer*^-P^* 2 | 517 | 1.7 | 1,264 | 4.1 | 6,526 | 21.1 | 67 | 0.22 | 53 | 0.17 |
| fertilizer*^-P^* 3 | 538 | 1.9 | 1,330 | 4.6 | 7,282 | 25.4 | 105 | 0.37 | 57 | 0.20 |

^a^ – calculated as ((*recA* reads)+(*atpD* reads·0.84)+(*gyrB* reads·0.44))/3, based upon a *recA* pHMM of 1,164 nucleotides, an *atpD* pHMM of 1,392 nucleotides and a *gyrB* pHMM of 2,618 nucleotides.

^b^ – calculated as (PHO reads·100)/length-normalized estimate of number of “genomes”

**Supplementary Table 2**. The number of metagenome reads with homology to the ubiquitous, single-copy genes *gyrB*, *recA* and *atpD* and the suite of PHO genes studied in this paper. The abundance of the single-copy genes was used to assess the total number of notional genomes associated with each dataset. The relative abundance of each PHO gene was then expressed as a proportion of this estimate.
